# Phytochemical Profile and Biological Activities of Rtanj’s *Hypericum perforatum* Infusion Tea and Methanolic Extracts: Insights from LC-MS/MS and HPTLC–Bioautography

**DOI:** 10.3390/plants14091377

**Published:** 2025-05-01

**Authors:** Sofija Kilibarda, Marko D. Jović, Danijel D. Milinčić, Sandra Vuković, Jelena Đ. Trifković, Mirjana B. Pešić, Aleksandar Ž. Kostić

**Affiliations:** 1 Department for Crop and Vegetable Production, Faculty of Agriculture, University of Belgrade, Nemanjina 6, 11080 Belgrade, Serbia; sofija.kilibarda@agrif.bg.ac.rs (S.K.); sandra.vukovic@agrif.bg.ac.rs (S.V.); 2 Innovation Center, Faculty of Chemistry, University of Belgrade, Studentski Trg 12-16, 11000 Belgrade, Serbia; markojovic@chem.bg.ac.rs; 3 Department for Chemistry and Biochemistry, Faculty of Agriculture, University of Belgrade, Nemanjina 6, 11080 Belgrade, Serbia; danijel.milincic@agrif.bg.ac.rs (D.D.M.); mpesic@agrif.bg.ac.rs (M.B.P.); 4 Faculty of Chemistry, University of Belgrade, Studentski Trg 12-16, 11158 Belgrade, Serbia; jvelicko@chem.bg.ac.rs

**Keywords:** St. John’s Wort, tea herba, spectrophotometric analysis, chromatographic analyses, phenolic compounds, antibacterial activity, antioxidant activity

## Abstract

This study aimed to examine wild-growing *Hypericum perforatum* L. tea (*Hyperici herba*) collected from Rtanj Mountain (Serbia). This research includes the following approaches: phytochemical and antioxidant characterization of *H. perforatum* infusion tea to determine its realistic composition (What do we consume when drinking the tea?), as well as a detailed examination of methanol(ic) extracts as the optimal extraction system. Due to the broad spectrum of both polar and nonpolar metabolites, 80% methanolic and pure methanol extracts were prepared for ultra-high-performance liquid chromatography coupled with quadrupole time-of-flight mass spectrometry (UHPLC Q-ToF) characterization through untargeted metabolomics analysis. Given the high diversity of compounds identified, the 80% methanolic extract was selected for further antioxidant examination and bioautographic characterization, including an antimicrobial activity assessment. UHPLC Q-ToF analysis identified 35 phenolics in the methanolic extract, compared to 25 metabolites in the infusion tea. The main differences were observed in flavonol/flavan-3-ol aglycones, xantones, and coumestans, which are more nonpolar compounds found only in the methanol(ic) system. Notably, specific *H. perforatum* metabolites were entirely absent in the infusion tea. Specifically, pseudohypericin, pseudoprotohypricin, and adhyperfirin were detected in the pure methanol extract, whereas hyperfirin was present in both methanol(ic) extracts. Additionally, eight furano-polycyclic polyprenylated acilphloroglucinols (FPPAPs) were identified in the methanol(ic) extracts as possible products of the thermal degradation and/or oxidation of hypericin/hyperforin. Both the infusion tea and methanolic extracts exhibited excellent antioxidant properties, with variations depending on the applied assay. High-performance thin-layer chromatography (HPTLC) analysis also confirmed the presence of a wide spectrum of phytochemical classes. Bioautography confirmed a promising activity of methanolic extracts against both *Staphylococcus aureus* and *Klebsiella pneumoniae*.

## 1. Introduction

**Figure 1:**
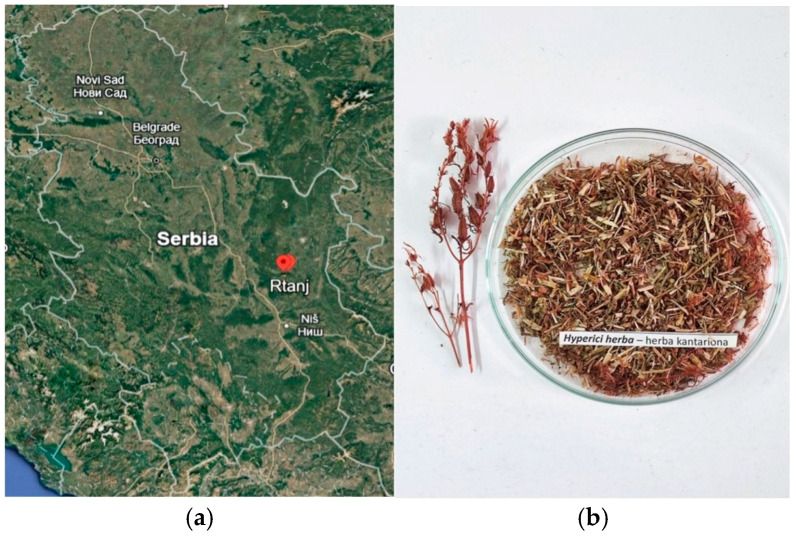
(**a**) Location of Rtanj Mountain; (**b**) *Hyperici herba*.

Rising from the plains of eastern Serbia, Rtanj Mountain (Figure 1a) captivates with its distinct pyramid-like shape and rich natural heritage. Its unique geographical position, varied microclimates, and fertile terrain have fostered abundant plant life, making it one of the most notable ecosystems in the region. The mountain is also renowned for its exceptional biodiversity, serving as a habitat for a wide array of plant species, many of which are endemic, rare, or relict [1,2]. Beyond its ecological importance, Rtanj holds a revered place in traditional medicine and herbalism [3]. Known for its abundance of medicinal plants, the region has been a vital source of natural remedies for generations. Local communities have long relied on these natural resources to treat ailments ranging from digestive disorders to respiratory conditions [2,3]. Traditional knowledge of herbal medicine has been passed down through generations, blending ancient practices with modern understanding [4]. Today, Rtanj remains a focal point for herbalists and researchers alike, drawn to its rich array of natural remedies. The mountain stands as a sanctuary for medicinal plants, emphasizing its cultural and practical importance in connecting the abundance of nature with human health and well-being.

Ethnobotanical research by Zlatković et al. [2] highlights *Hypericum perforatum* L. as the most cited medicinal plant in Rtanj, underscoring its great importance in the region’s traditional healing practices. Furthermore, its most common use involves the aerial parts of the plant with inflorescences (*Hyperici herba*) collected from May to June and prepared as an infusion. This vibrant herb, commonly known as St. John’s Wort, has long been valued for its diverse therapeutic properties, including its use as a sedative, anti-inflammatory agent, and treatment for respiratory and urogenital tract conditions, hemorrhoids, and burns [4,5,6]. In addition, this perennial plant has garnered significant scientific interest for its chemistry, pharmacology, and clinical properties, particularly its antidepressant effects. Moreover, the literature has illuminated its ability to promote relaxation, enhance cognitive function, and delay the onset of rapid eye movement (REM) sleep—a hallmark of many conventional antidepressant drugs—further emphasizing *Hypericum’s* potential as a natural therapeutic agent [7,8]. The plant’s pharmacological properties come from its complex phytochemical composition, with hyperforin and hypericin identified as its primary active constituents, complemented by phenolic compounds such as flavonoids and tannins. Brondz et al. [9] reported the remarkable potential of hyperforin as both an antibiotic and an immunomodulatory agent. It enhances phagocytosis and bacterial breakdown by human polymorphonuclear neutrophils, exhibits activity against antibiotic-resistant Gram-positive and several Gram-negative pathogens, and can cross critical physiological barriers such as the blood–brain barrier, making it particularly valuable for treating meningitis and gonorrhea in immunocompromised patients, including those with AIDS.

Therefore, this study aimed to analyze the phytochemical profile and assess the antioxidant properties of *H. herba* (prepared from wild flora sourced from the pristine Rtanj Mountain region, Figure 1b) as infusion tea, as well as in the form of methanol(ic) extracts. The goal was to compare extraction efficiency and bioactivity under realistic conditions (infusion tea) and maximal extraction conditions (methanol extracts). Additionally, HPTLC bioautography was performed followed by an antibacterial activity assessment of a representative methanolic extract. This research aims to highlight the diversity of bioactive compounds and the therapeutic potential of *H. perforatum*, which is rooted in the rich biodiversity and traditional knowledge of the area.

## 2. Results

### 2.1. Proximate Phytochemical Composition

Spectrophotometric analysis was conducted to determine the content of bioactive compounds in *H. perforatum* infusion tea (hereafter referred to as the aqueous extract) and methanolic extracts. Absorbance measurements were recorded across relevant wavelengths to identify corresponding phenolic compounds, including hydroxycinnamic acid derivatives, and tannins (Table 1). The total phenolic content (TPC) was 26.48 mg/g GAE dry weight (DW) for aqueous extracts and 31.38 mg/g GAE DW for methanolic extracts, with the latter showing a statistically significant higher value. Additionally, the total hydroxycinnamic acid derivatives (HCAs) were quantified at 3.28 mg/g CGAE DW for aqueous and 4.22 mg/g CGAE DW for methanolic extracts, while the total tannin content was 12.83 mg/g TAE and 7.51 mg/g TAE DW for aqueous and methanolic extracts, respectively. Statistically significant differences among solvents were observed for HCA analysis, whereas no significant differences were found for tannin content. These findings indicate that *H. herba* is a potent source of phenolics.

### 2.2. UHPLC-QToF-MS Analysis of Phenolic Compounds

To obtain a comprehensive and precise phytochemical profile of the examined tea infusion and the methanolic extract, a powerful and advanced ultra-high-performance liquid chromatography coupled with quadrupole time-of-flight mass spectrometry (UHPLC Q-ToF MS) analysis was performed. The MS base peak chromatograms (Figure S1) and base peak chromatograms of precursor ions and their MS/MS fragments (product ions) (Figure S2) in negative and positive ionization modes of *Hypericum perforatum* aqueous infusion tea and methanolic extracts are depicted in the Supplementary Material. The peaks of all identified compounds were extracted from MS base peak chromatographs (Figure S1), based on monoisotopic masses of their precursor ions. The profile of phenolic compounds is presented in Table 2. In total, an untargeted analysis identified 35 phenolic compounds in the methanolic (MW) extract and 25 phenolic compounds in the aqueous tea infusion (ATI). The results confirmed that MW was the optimal solvent for extracting *H. perforatum* phenolic compounds. Based on their similar chemical structure, all identified compounds were classified into six subgroups: (1) hydroxybenzoic acids and glycosides; (2) hydroxycinnamic acid derivatives (glycosides and esters); (3) flavan-3-ols and procyanidins; (4) flavonol aglycones and glycosides; and (5) other flavonoids. It was observed that the MW extract contained thirteen flavonol derivatives, making them the most diverse class of phenolics, followed by nine hydroxycinnamic acid derivatives, seven hydroxybenzoic acids and derivatives, two flavan-3-ols, and one procyanidin B-type dimer. In contrast, the aqueous extract, prepared as infusion tea, had the highest diversity of phenolic acids (seventeen hydroxycinnamic and hydroxybenzoic acids and their derivatives), surpassing the methanolic extract in this category. However, the presence of flavonoids and flavonoid-like compounds in this extract was significantly lower, as expected, with no flavan-3-ols, flavanone, and flavones, most probably due to polarity differences. Individually, hydroxybenzoic acid aglycones—including hydroxybenzoic acid, dihydroxybenzoic acid, and gallic acid—were confirmed in both extracts (MW and ATI), along with dihydroxybenzoic acid hexoside II and vanillic acid hexoside. Other detected hydroxybenzoic acid glycosides were selectively present in the extracts. Thus, gallic acid hexoside isomers and dihydroxybenzoic acid hexoside I were confirmed in ATI, while syringic acid hexoside and dihydroxybenzoic acid hexoside III were detected in the MW extract. These hydroxybenzoic acid derivatives were identified based on their exact mass and typical fragments obtained through the loss of CO_2_ (−44 Da) for aglycones and hexosyl moiety (−162 Da) for glycosides. Hydroxycinnamic acid derivatives were mostly confirmed in the form of esters with quinic acid, or more rarely, in the form of glycosides. Various isomers of caffeoylquinic (except isomer IV) (compounds **15**–**17**) and *p*-coumaroylquinic (compounds **12** and **13**) acid were detected in both extracts (MW and ATI). However, feruloylquinic acid and dicaffeoylquinic acid were confirmed only in the MW extract. Hydroxybenzoic acid derivatives with quinic acid were identified based on their typical fragments, originating from quinic acid (191, 173, and 155 *m*/*z*), caffeic acid (179, 161, and 135 *m*/*z*), coumaric acid (163 and 119 *m*/*z*), and ferulic acid (193, 149, and 134 *m*/*z*). Hexosides of coumaroylquinic and caffeoylquinic acid, as well as rosmarinic acid, were confirmed only in the aqueous tea infusion. Dicaffeoylquinic acid (compound **22**) and caffeoylquinic acid hexoside (compound **23**) had similar MS fragmentation but distinct exact masses and formulas, which contributed to their identification. Hydroxycinnamic acid glycosides were selectively found in each extract. Coumaric acid hexoside was confirmed in the ATI, while caffeic acid hexoside was detected in the MW extract.

Among flavonoids, flavanols and flavan-3-ols were predominant and primarily identified in the extracts. Catechin and epicatechin were the only detected monomeric flavan-3-ols, along with one procyanidin B-type dimer, identified as procyanidin B1. Catechin and epicatechin are isomers that have the same exact mass and MS fragments but different retention times, and their identification was confirmed using available standards. These isomeric compounds were found only in the MW extract but not in the tea infusion. However, the procyanidin derivative was detected in both aqueous and methanolic extracts. Flavonols were detected in the form of aglycones, glycosides, and acyl derivatives. Flavonol aglycones (compounds **27**–**31**) were identified based on their typical MS fragments, generated through retro Diels–Alder cleavage of the heterocyclic C ring [10]. These aglycones were found only in the MW extract. Quercetin glycosides (compounds **33**–**35** and **39**) and acyl derivatives (compounds **37** and **38**) were predominantly found in both extracts, except quercetin 3-*O*-pentoside, which was detected only in the MW extract. All quercetin derivatives exhibited typical MS fragments at 300 *m*/*z* ([Y_0_–H]^–^) and 301 *m*/*z* (Y_0_^–^) (deprotonated quercetin aglycone) obtained through *O*-glycosidic bond cleavage, as well as fragments at 151 *m/z* (^1,3^A^–^) and 179 m/z (^1,2^A^–^) derived from quercetin aglycone. 

**Figure 2:**
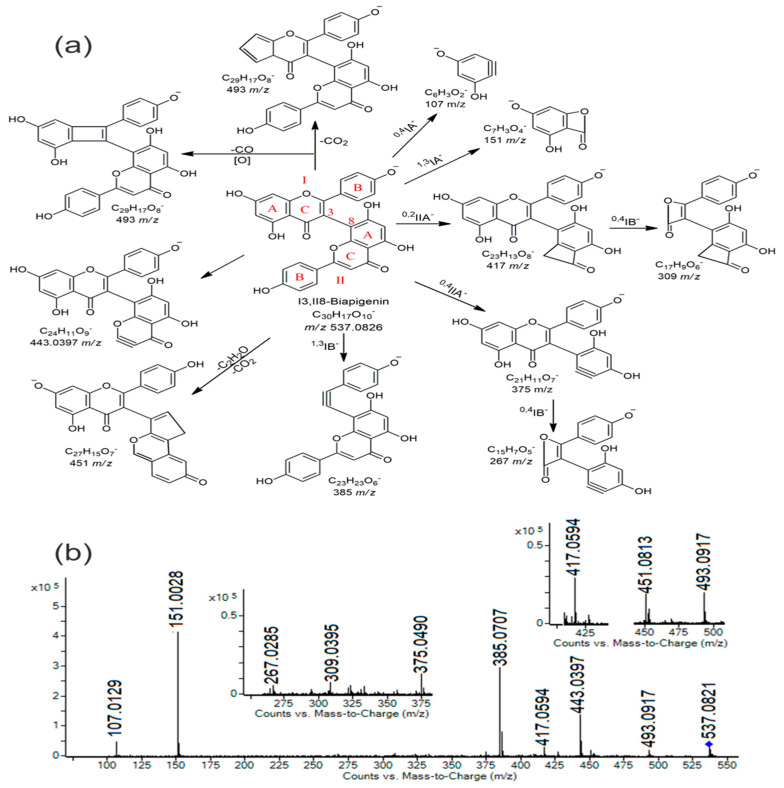
(**a**) Proposed fragmentation pathway with typical fragments; and (**b**) MS/MS fragmentation pattern of I3,II8-biapigenin.

Apart from the quercetin derivative, myricetin 3-*O*-hexoside was also detected, but only in the MW extract. In addition to this, three other flavonoids were identified and confirmed exclusively in the MW extract: naringenin (a flavanone), trimetoxyflavone (similar to salvigenin), and I3,II8-biapigenin (a flavone). The biflavone recognized as I3,II8 biapigenin is a typical phenolic compound derived from *H. perforatum* [11,12]. This compound has a unique structure compared to other biflavones in the literature [13] featuring specific C_3_-C_8_ linkage and characteristic fragments obtained through retro Diels–Alder cleavage of the heterocyclic C ring (Figure 2a) [12,13]. The characteristic MS/MS fragmentation pattern (MS/MS spectra) of I3,II8-biapigenin is presented in Figure 2b.

### 2.3. UHPLC Q-ToF MS Analysis of Other (Non-Phenolic) Bioactive Compounds

In addition to phenolic compounds, *H. perforatum* was a rich source of other bioactive compounds, such as naphthodianthrones, polycyclic polyprenylated acylphloroglucinols (PPAPs), xanthones, and coumestans. The putatively identified compounds from these classes of biomolecules are presented in Table 3.

These bioactive compounds were completely absent from the aqueous tea infusion, as expected. For this reason, the methanolic (methanol/water; MW) extract and the less polar pure methanol (M) extract were analyzed to obtain the best possible characterization of the bioactive compounds mentioned. Nevertheless, after UHPLC-QToF-MS analysis of the methanol(ic) extracts, the absence of two well-known *H. perforatum’s* bioactive compounds—hypericin and hyperforin—was observed. However, other naphthodianthrones and PPAPs were confirmed, in accordance with data from the literature. Pseudohypericin and pseudoprotohypericin were found only in the M extract and were the only detected molecules from the naphthodianthrone group.

Polycyclic polyprenylated acylphloroglucinols (PPAPs) are widely represented and are characteristic compounds of *Hypericum* spp. The well-known PPAPs, hyperforin and adhyperforin, were not found in the analyzed extracts. However, hyperphyrin, adhyperphyrin, and various furano-PPAPs derivatives (PPAP fused to a partly reduced furan ring at C_3_–C_2_–O_2_ (type A) or C_1_–C_2_–O_2_ (type B)) (compounds **47**–**54**, Table 3) were confirmed in the M extract. Additionally, some of these compounds (**50** and **52**–**54**) were selectively found in the MW extract, while none were detected in the tea infusion. The tentative identification of these compounds (in the absence of standards) was based on their exact mass (monoisotopic mass), MS fragmentation, and previously published nuclear magnetic resonance (NMR) [20,21,22,23,24] and mass spectrometry data [29,30,31] related to *H. perforatum*. References regarding the previous identification of these compounds in *Hypericum* spp., especially *H. perforatum,* are listed in Table 3.

Xanthones are primarily found in the roots of *H. perforatum* [16,25]. In this study, four typical *Hypericum* xanthones were confirmed: tetrahydroxyxanthone (similar to norathyriol), two prenylated tetrahydroxyxanthone (prenyl-tetrahydroxyxanthone and γ-mangostin), and a xanthone C-glycoside (mangiferin). All identified xanthones were confirmed in the MW extract. Additionally, tetrahydroxyxanthone and mangiferin were detected in the M extract, whereas no xanthone derivatives were confirmed in the tea infusion. In addition to these bioactive compounds, one coumestan, known as wedelolactone, was also confirmed in both methanol (MW and M) extracts. These compounds (xanthones and coumestan) were identified based on their monoisotopic mass and typical MS fragments, as well as confirmed by literature data [17,25,26,28].

### 2.4. Antioxidant Properties

The antioxidant properties of *H. perforatum* extracts were evaluated using multiple in vitro assays, demonstrating strong antioxidant potential (Table 4). For this purpose, ATI and MW extracts were used. The ABTS^⦁^⁺ scavenging capacity was higher in the aqueous extract, measuring 176.48 μmol/g Trolox, compared to 130.49 μmol/g Trolox in the methanolic extract. In contrast, the DPPH^⦁^ assay showed the opposite trend, with the methanolic extract exhibiting greater activity (149.99 μmol/g Trolox) than the aqueous extract (132.96 μmol/g Trolox). In both assays, the differences between solvents were statistically significant. The methanolic extract had higher values for the total antioxidant capacity determined via the phosphomolybdenum assay (TAC) (32.31 mg/g AAE), with statistically significant differences compared to the aqueous extract (20.73 mg/g AAE). Additionally, ferric reducing power (FRP) and cupric reducing antioxidant capacity (CUPRAC) were measured. The aqueous extract showed values of 21.08 mg/g AAE for FRP and 27.50 mg/g AAE for CUPRAC, while the methanolic extract exhibited higher values of 30.58 mg/g AAE and 20.06 mg/g AAE, respectively. Statistical analysis confirmed significant differences among solvents for both assays. These results suggest that the aerial flowering parts of St John’s Wort possess notable antioxidant capacity, likely due to their high phenolic and flavonoid content.

### 2.5. HPTLC Fingerprinting and Chemical Profile of H. Perforatum Methanolic Extract

**Figure 3:**
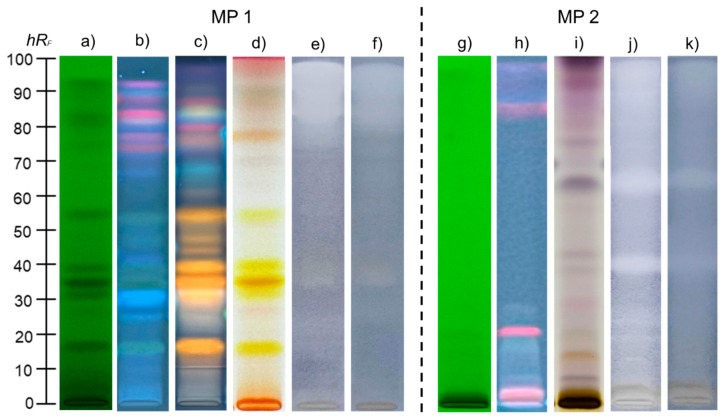
HPTLC fingerprints of *H. perforatum* methanolic extract developed using two mobile phases: MP 1 (ethyl acetate/toluene/formic acid/water, 16:4:3:2, *v/v/v/v*) and MP 2 (toluene/ethyl acetate/methanol, 55:40:5, *v/v/v*). Visualization was performed under UV 254 nm (**a**,**g**); UV 366 nm (**b**,**h**); UV 366 nm, after derivatization with NP/PEG reagent (**c**); white light, after derivatization with ASA reagent (**d**,**i**); white light, after MTT antibacterial assay against *S. aureus* (**e**,**j**); white light, after MTT antibacterial assay against *K. pneumoniae* (**f**,**k**).

Further characterization and identification of secondary metabolites of *H. perforatum* were performed using the methanolic extract, as it was confirmed to be the most efficient solvent in terms of the diversity and polarity of the extracted compounds. For this purpose, high-performance thin-layer chromatography (HPTLC) was employed, and the obtained results are presented in Figure 3a–d,g–i.

The chromatographic fingerprint profiles of *H. perforatum* extract were developed using two mobile phases (MP 1 and MP 2) with distinct polarities. By expanding the polarity range, HPTLC analysis enabled a more comprehensive chemical profiling. Chromatograms observed under UV 254 nm revealed a higher number of dark zones with MP 1 (Figure 3a), whereas chromatograms developed with MP 2 displayed fewer and less pronounced dark zones, corresponding to compounds with conjugated double bonds (Figure 3g). Under UV 366 nm, fluorescent zones in shades of blue, red, and gray were observed (Figure 3b,h). Similar to UV 254 nm, the chromatogram obtained with MP 1 yielded a greater number of fluorescent zones. For compounds not visible under UV light, derivatization with the ASA reagent was performed, revealing color variations from light gray and blue to pale violet, aiding in the differentiation of natural product classes. Using MP 2, the chromatogram exhibited blue and violet zones (Figure 3i), whereas ASA derivatization of the chromatogram obtained with MP1 revealed yellow to orange zones (Figure 3d).

### 2.6. HPTLC Antibacterial Activity of H. perforatum Methanolic Extract

Antibacterial activity against *S. aureus* and *K. pneumoniae* was detected by coupling HPTLC with the MTT colorimetric bioassay. The obtained results are presented in Figure 3e,f,j,k. HPTLC plates containing separated components of the *H. perforatum* methanolic extract were exposed to bacterial suspensions and incubated, allowing bacterial growth directly on the plates. Zones corresponding to antibacterial activity in the extract appeared as light-yellow spots against a blue-violet background. The chromatogram obtained with MP 1 revealed an intensely active region from *hR_F_* 80 to the front of the mobile phase, where less polar compounds are concentrated, along with a weaker active zone at *hR_F_* 35 (Figure 3e,f). In contrast, MP 2 facilitated more efficient separation of the lipophilic fraction, which was concentrated at the front of the MP 1 chromatogram. The MP 2 chromatogram showed intense antibacterial zones at *hR_F_* values of 40 and 65 (Figure 3j,k).

The obtained chromatograms for bacterial strains were further analyzed to quantify antibacterial activity using an image processing technique. Specifically, the areas of peaks corresponding to active zones were processed with ImageJ software 2.16.0, enabling a comparison of the extract’s antibacterial activity with that of streptomycin, a reference standard to which both tested strains are sensitive. The peak areas of each active zone (expressed in pixels) were summed to represent the overall antibacterial activity of the extract. According to summed peak areas and calibration curves, the activity of the extract was expressed as milligrams of streptomycin equivalent (StrpE) per milliliter of the tested extract. Streptomycin was applied to the HPTLC plates under the same experimental conditions, and standard curves for streptomycin were generated by plotting the applied amount (μg) against the corresponding peak areas measured in pixels. Validation parameters for the standard curve are summarized in Table A1 (Appendix A), while the obtained antibacterial activity, expressed as streptomycin equivalents (StrpE), is presented in Table 5.

As shown in Table 4, the analysis revealed greater antibacterial activity against both tested strains when MP 2 was applied. In addition, *S. aureus* was more susceptible to the activity of *H. perforatum* methanolic extract.

## 3. Discussion

### 3.1. Spectrophotometric Characterization of H. perforatum Tea Infusion and Methanolic Extract

The obtained spectrophotometric results provide important insights into the proximate phytochemical composition and antioxidant capacity of *H. perforatum* infusion tea and methanolic extract from Rtanj, contributing to a broader understanding of its bioactive potential and relevance as a natural source of antioxidants. The diverse phenolic compounds of this plant species are well known for their wide range of therapeutic properties, such as antidepressant, antiviral, antibacterial, photosensitizing, and antioxidant effects [32]. Additionally, the accumulation of phenolic compounds in plants is significantly influenced by environmental factors, including abiotic stresses such as temperature, drought, salinity, and UV radiation, as well as biotic factors such as pathogen attacks and herbivory. These stressors activate the plant’s metabolic pathways as a defense mechanism [33]. This suggests that *H. perforatum* from spontaneous flora may serve as a richer source of phenolics compared to cultivated varieties due to its exposure to natural stressors in its native habitat. The increased TPC in the methanolic extract, compared to the aqueous extract, can be attributed to methanol’s enhanced ability to penetrate plant cell membranes, facilitating the efficient release of cellular compounds and yielding a higher crude extract [34]. In the study by Tahirović et al. [35], St. John’s Wort infusion tea extract exhibited a total phenolic content (TPC) of 274.5 mg GAE/100 mL. In contrast, the present study, which analyzed aqueous extracts from wild flora, measured a TPC of 26.48 mg/g GAE. Moreover, water extracts from dried *H. perforatum* cultivated in Iran exhibited significantly lower total phenolic content measured at 0.051 mg/g GAE [36]. This prominent difference may be attributed to the geographical origin and unique characteristics of the spontaneous flora of Rtanj Mountain, as well as specific biotic and abiotic factors at the collection sites. Other influencing factors include cultivation practices, agronomical conditions, and the extraction method, all of which can affect the synthesis and yield of these compounds. Furthermore, Krivokapić and Pejatović [37] concluded that the TPC and TFC contents of St. John’s Wort extracts are influenced by the type of extraction solvent, altitude of the habitat, and varying environmental conditions. Their findings also highlighted irregularities in the dynamics of these compounds throughout the vegetation cycle [37]. Nevertheless, Alahmadi et al. found that water extracted the highest TPC among methanol and ethanol solvents, likely due to the strong hydrogen bonding ability of phenolic compounds with water, which enhances their solubility in this polar solvent [38,39]. Although Table 1 shows that hydroxycinnamic acids derivatives (HCAs) are present in lower concentrations, their significance remains substantial. These substances are valued for their potent antioxidant properties, which help prevent oxidative stress-related diseases, including heart-related and progressive brain disorders, as well as cancer [39]. Additionally, several HCAs exhibit anti-inflammatory and antimicrobial activities, further supporting their therapeutic potential, while their derivatives are emerging as a promising class of natural compounds for managing lipid metabolism and obesity [40,41]. Moreover, the antimicrobial properties of this plant species are largely attributed to its tannin content. These compounds bind to bacterial adhesins, disrupting the availability of receptors on bacterial cell surfaces and thereby preventing the bacteria from adhering and infecting host tissues [42]. While flowers serve as the primary storage organs for hypericin and hyperforin—two of the most studied bioactive compounds of this herbaceous plant, the leaves are significantly richer in tannins [43]. This highlights the importance of utilizing the entire herb, particularly the leaves, as tannins contribute to the plant’s antioxidant and protective effects, reinforcing its medicinal value.

Due to the diverse nature of phytochemicals in plant water extracts, evaluating antioxidant activity requires multiple assays to provide a comprehensive understanding of the infusion’s free radical-neutralizing capacity. The results from antioxidant assays demonstrated significant potential, with the ABTS^•+^ assay exhibiting higher radical scavenging activity in aqueous extracts compared to DPPH^•^. This difference can be attributed to the distinct target molecules of the two assays. ABTS^•+^ assay is generally more responsive to compounds that can neutralize both lipophilic and hydrophilic radicals, whereas the DPPH^•^ assay primarily evaluates the scavenging ability against the DPPH radical, which may explain the lower observed activity [44,45]. Conversely, in the methanolic extract, the trend was reversed with the DPPH^•^ value exceeding that of ABTS^•+^, consistent with the findings of Kakouri et al. [46]. This reverse trend is somehow logical due to the lower polarity of methanol and its higher ability to extract “more” lipophilic compounds. Hernandez et al. [47] reported that water extracts of *H. perforatum* from five different suppliers exhibited antioxidant activity in the DPPH radical scavenging assay, with EC50 values ranging from 9.0 ± 0.2 to 18.4 ± 0.8 μg of dry extract/mL [48]. Similarly, Božin et al. [48] found that ethanolic extracts of the aerial parts of this species, collected from various locations in the Central Balkans, had IC50 values between 3.48 and 5.68 μg/mL for the same assay, indicating the influence of geographical and environmental factors. According to Radulović et al. [49], the total antioxidant capacity (TAC) of *Hypericum* extracts is primarily attributed to flavonoids, which effectively scavenge free radicals and protect against oxidative damage. The study also highlights that the TAC of methanolic extracts correlates with the flavonoid distribution across different plant organs, with variations in antioxidant activity influenced by the flavonoid content in the flowers, leaves, and stems. However, antioxidant capacity does not follow a consistent pattern, as it can vary based on species identity, as well as the site and harvesting time. The CUPRAC assay in the current study produced slightly higher values compared to both TAC and FRP assays. This can be attributed to the method’s advantages, such as the simplicity of pH adjustment, stability and availability of reagents, cost efficiency, and its ability to assess both lipophilic and hydrophilic antioxidants [50]. In the same study, the antioxidant capacity of wild-grown *H. perforatum* from Turkey was evaluated using the CUPRAC assay, comparing water extracts of the flowers and leaves; the flowers showed greater antioxidant properties. This observation aligns with the total phenolic content, which was consistent with the measured antioxidant capacity. Additionally, higher CUPRAC assay values were observed in methanolic extracts in the study by Ersoy et al. [51].

While the antioxidant activity of St. John’s Wort extracts is primarily attributed to flavonoid glycosides and phenolic acids, further research, especially in water-based solvents, is needed to deepen this understanding [32]. These results highlight the potential of Rtanj Mountain as a rich source of wild *H. perforatum*, with its tea offering promising antioxidant and therapeutic properties, making it a valuable natural remedy from the region.

### 3.2. UHPLC-QToF Characterization of Bioactive Compounds Derived from H. perforatum

To gain a comprehensive understanding of *H. perforatum’s* bioactive profile, three extraction systems were applied in this study, revealing significant differences in the obtained profiles. Methanol is widely recognized as one of the most powerful solvents in phytochemistry due to its moderate polarity, which enables the extraction of a broad spectrum of plant metabolites, including phenolics (both simple phenolic acids and complex flavonoids), alkaloids, certain terpenes, etc. [52]. Similar conclusions have been drawn in most recent reviews on *Hipericum* extraction abilities [53]. The phenolic compounds identified in this study align with previously reported phenolic profiles of *H. perforatum* [54,55]. Quercetin glycosides (guaijaverine, quercitrin, hyperoside, miquielianin, and rutin), quercetin 3-*O*-(6”-*O*-acetyl)hexoside, and I3,II8-biapigenin, as well as hydroxycinnamic acid derivatives with quinic acid (caffeoyl-, coumaroyl-, and feruloylquinic acid), are typical phenolic compounds originating from *H. perofratum* [11,27,56,57]. While an aqueous extract in the form of an infusion provides a more realistic representation of the phytochemicals available during consumption, the MW extract was significantly richer in identified phenolic compounds, particularly nonpolar flavonoids and their derivatives. Unlike the aqueous extract, which contained seven identified flavonols (mostly in the form of glycosides), the methanolic extract contained thirteen flavonols, including both aglycones and derivatives. Additionally, all flavonol aglycones and both flavan-3-ols (catechin and epicatechin) were identified exclusively in the methanolic extract. Conversely, the profiles of phenolic acids and their derivatives were more comparable across different extraction methods due to their solubility in both water and organic solvents. A very similar report was published for *H. perforatum* from the neighboring country, North Macedonia (Pelister Mountain), where the authors applied the same methanolic solvent (80%) in order to obtain the HPLC/DAD/ESI-MS phenolic profile [56]. In total, ten of the same compounds were reported including feruloylquinic and *p*-coumaroylquinic acids, catechin, epicatechin, procyanidin B-type dimer, hyperoside, quercetin, rutin, and 3,8-biapigenin, as well as quercetin-3-*O*-pentoside (quercetin-3-*O*-arabinoside or guaijaverin) in the Macedonian *H. perforatum*. Similarly, a previous study on *H. perforatum* infusions from Northern and Eastern Serbia [58] used UHPLC-DAD-ESI-MS/MS to determine the phenolic profile. Due to the geographical proximity, the obtained results were even more comparable, with fifteen shared compounds identified. Unlike the Macedonian study, infusions from Serbian *H. perforatum* contained naringenin and kaempferol glycosides (whereas in the present study, these compounds were confirmed as aglycones), as well as quercitrin and an acetylated quercetin derivative, both of which were also confirmed in this study [58]. I3,II8 biapigenin is quite an interesting metabolite, identified exclusively in the methanolic extract. This compound has demonstrated significant pharmacological potential, with proven antidepressant and neuroprotective activity [58]. While studies indicate that it can enter the bloodstream in mice, it does not appear to penetrate brain tissue [12].

**Figure 4:**
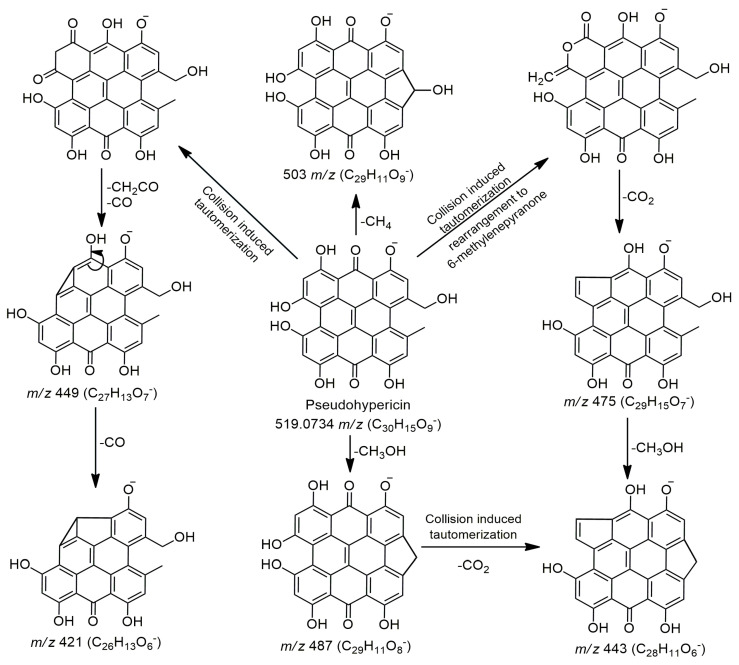
Proposed fragmentation pathway of pseudohypericin.

Apart from phenolic compounds, no other bioactive compounds were found in the aqueous tea infusion. This was expected, as these compounds are generally insoluble or only slightly soluble in water due to their complex nonpolar structures. Naphthodianthrones are mostly accumulated in oil glands, flowers (particularly in pistils), and leaves [18,59]. This was confirmed by previous studies which found that neither hypericin nor hyperforin was present in root samples but only in flower shoots of Macedonian *H. perforatum* [56]. In this study, pseudohypericin and protopseudohypericin were the only detected naphthodianthrones, and they were found exclusively in the methanol (M) extract. These compounds were identified based on their exact mass and available MS data from the literature. Applied ionization conditions (fragmentor energy of 175 V and collision energy CE = 30 eV) have given scarce MS fragmentation of detected naphthodianthrones and the highest intensity for precursor ions (*m*/*z* 519 or 521), while all fragment showed low intensities (Figure S3, characteristic MS/MS fragmentation pattern of pseudohypericin). However, data related to the mass spectrometric characterization of these compounds are scarce, and only a few studies provide explanations regarding the formation of the majority fragments obtained by eliminating CH_2_CO (ketene), CO, and/or CO_2_ [14,15]. The most acceptable explanation for this MS fragmentation involves rearrangement to a tautomeric species and/or conversion to a 6-methylenepyranone anion, as shown in the example of pseudohypericin (Figure 4**)**. The fragment at 487 *m*/*z* was the main and the most intensive fragment obtained by MS fragmentation of pseudohypericin, followed by fragments at 475 and 503 *m/z* (Figure S3). The fragment at 475 *m*/*z* was obtained by rearrangement and elimination of CO_2_. On the other hand, fragments at 503 and 487 *m*/*z* are the most likely independently formed ones, in one step of MS fragmentation of pseudohypericin, but the loss of CH_3_OH (487 *m/z*) is obviously preferable/favorable to the loss of CH_4_ (503 *m*/*z*).

Pseudohypericin and protopseudohypericin were also identified by Tusevski et al. [17] in *H. perforatum* hairy roots. In addition, Rašković et al., [19] showed the predominant presence of these compounds in ethanolic extracts, while only traces were detected in some aqueous extracts. Interestingly, hypericin was not detected in the analyzed extracts. This can be explained by the fact that pseudohypericin is the predominant naphthodianthrone in *H.perforatum*, with 2–4 times higher content than hypericin [16,19,27]. Moreover, hypericin is an unstable compound, easily degradable, and sensitive to light, temperature, and humidity [60,61]. Thus, different growing conditions and processing of *H. herba* could also contribute to the absence of hypericin in the studied extracts.

**Figure 5:**
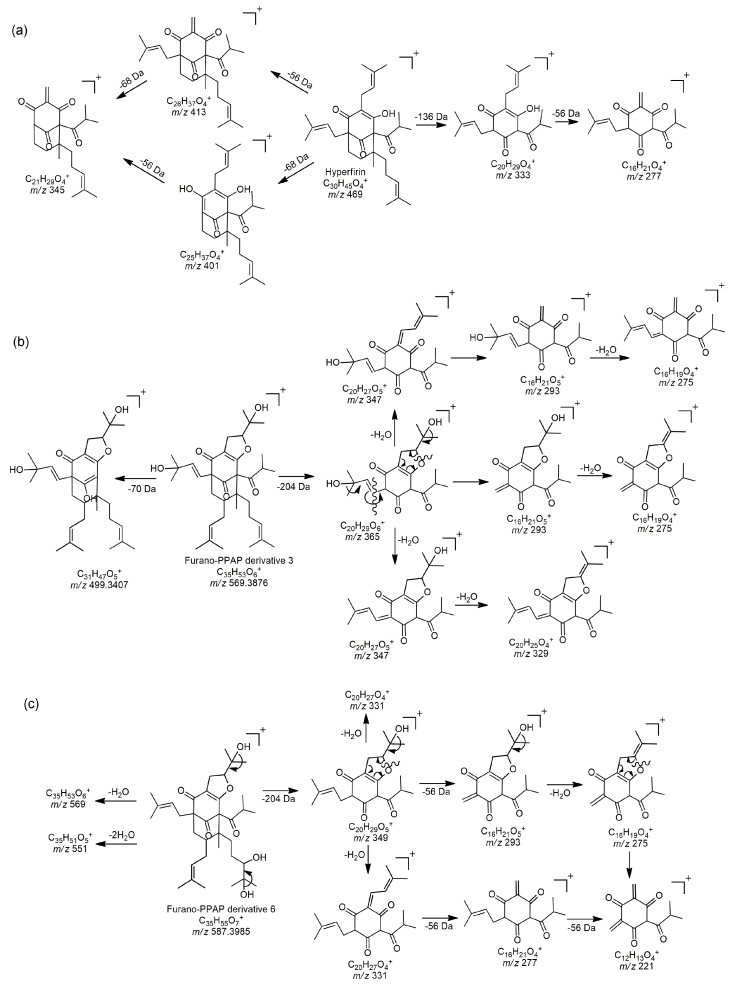
Proposed fragmentation pathways of (**a**) hyperfirin; (**b**) FPPAP derivative 3 (compound like Hyperformitin C or Hyperformitin D); (**c**) FPPAP derivative 6 (compound like Hyperidione F).

Hyperfin, adhyperphyrin, and various furano-PPAPs were detected in the methanol (M) extract, with some also present in the methanolic (MW) extract, while they were entirely absent in the tea infusion. This was expected, as PPAPs are lipophilic compounds that are either insoluble or only slightly soluble in aqueous solutions [16]. Hyperforin and adhyperforin were not detected in the analyzed extracts. These compounds are highly sensitive and degrade quickly when exposed to heat and light [29,60]. Although hyperforin and adhyperforin are well-known PPAPs from *Hypericum* spp., Rašković et al. [19] showed that hyperfirin and adhyperfirin predominate in *H. perforatum*, with significantly higher concentrations. In this study, various furano-PPAPs were detected. Compounds of this type were previously characterized by NMR and reported in *H. perforatum* [20,22,23,24]. The prenyl chain can cyclize and form furano-PPAP (FPPAPs) derivatives. Due to the large number of isomers (structural isomers and stereoisomers), it is often difficult to predict the exact structures of the identified FPPAPs, as well as the position and isomeric form of their side chains (prenyl chain, butenyl chain, dimethylketene, etc.). The identification and proposed fragmentation pathways of hyperphyrin, as well as two furano-PPAP derivatives (compounds **49** and **52**, previously reported in the literature), are presented in Figure 5a–c, in accordance with the available literature data related to MS fragmentation of structurally closest compounds [29,30,31,62]. The characteristic MS/MS fragmentation patterns (MS/MS spectra) of these compounds are presented in Figure S4a–c. All furano-PPAPs exhibited the most intense fragments at 293 *m/z*, followed by a fragment at 275 *m/z*. Fragments at 365, 347, 329, 349, and 331 *m/z* were selectively observed, depending on the structure of the side chains, as shown in Figure 5b,c. Taking into account the MS fragments, it can be clearly observed that some compounds belong to FPPAPs; however, these compounds have not been previously reported in the literature and are labeled as unknown FPPAP derivatives. For this reason, further NMR or similar analyses are necessary to confirm or fully identify these compounds. Finally, the PPAPs detected in this study have shown antibacterial activity and wound-healing ability, as well as antidepressant, immunosuppressive, enzyme inhibitory, and anticarcinogenic potential [20,22,57].

Xantones and coumestans were identified in the methanolic (MW) extract and selectively confirmed in the methanol (M) extract due to their complex nonpolar structures. Xanthones are mainly found in the roots; however, since this study analyzed the whole *H. herba*, their presence in the extracts is justified. Xanthones exhibit a broad range of pharmacological properties, including antioxidant, antimicrobial, cytotoxic, vascular, antimicrobial, antidiabetic, and hepatoprotective activities [17,63]. Notably, the presence of wedelolactone in the methanol(ic) extracts is very interesting. According to a literature review, this coumestan-type compound has recently gained significant attention due to its diverse pharmacological properties, including potential anticancer, anti-inflammatory, antidiabetic, and antimyotoxic activities [64]. Additionally, it has demonstrated the ability to fight against obesity, and it has exhibited hepatoprotective, cardioprotective, pulmonary, and dental protective activities [64]. These attributes make wedelolactone a compound of particular interest in modern pharmacology. However, to date, this compound has been predominantly reported in plants belonging to the Asteraceae family, particularly in the *Wedelia* and *Eclipta* genera [64]. To our knowledge, there is only one previous report of wedelolactone presence in *H. erectum* from China, where it was confirmed using column chromatography and recognized as the carrier of the plant’s antihemorrhagic activity [28]. Based on this, it can be speculated that this study represents the first report of wedelolactone in *H. perforatum herba*. Although there are previous studies on the phytochemical fingerprint of *H. perforatum* from Serbia and the surrounding region, this study provided a more in-depth analysis, offering novel insights into the obtained results. This is largely due to the great diversity of phytochemicals present in *H. perforatum*; these compounds are quite different in terms of their chemical structures and belong to distinct groups of compounds. Therefore, it is nearly impossible to extract all of them with one solvent system for analysis by some specific techniques, in this case, UHPLC. Thus, in this study, three solvents with different polarity were applied, providing us with a thorough and comprehensive analysis of the wide spectrum of *H. perforatum*’s bioactive compounds including all specific metabolites.

### 3.3. HPTLC Phytochemical Fingerprinting of Methanolic H. perforatum Extract

Two mobile phases of different polarity were applied to analyze compounds of varying polarity present in the methanolic *H. perforatum* extract. The first mobile phase, composed of ethyl acetate/toluene/formic acid/water, 16:4:3:2, *v/v/v/v* (MP 1, more polar), was suitable for separating polar metabolites due to its higher proportion of polar components relative to nonpolar ones. In contrast, the second mobile phase, consisting of toluene/ethyl acetate/methanol, 55:40:5, *v/v/v* (MP 2, more nonpolar), with toluene as the dominant nonpolar solvent, was better suited for separating less polar compounds. Visualization of the separated compounds was performed by observing their coloration, fluorescence under UV light, and appearance after derivatization (Figure 1). Before derivatization, by exposing the plates to UV light at 254 nm, aromatic compounds and those with conjugated double bonds appeared as dark spots against the green fluorescence background of the chromatographic plate, due to their absorption of UV light and quenching of fluorescence of the indicator on the plate [65]. The higher number of dark zones observed with MP1 under UV 254 nm was consistent with its reported suitability for phenolic compound separation [66]. The observed fluorescent zones under 366 nm corresponded to compounds whose fluorescence is excited by long-wavelength UV light [65]. The ability of MP1 to produce a higher number of fluorescent zones further confirmed its effectiveness in separating phenolic compounds, which typically appear as fluorescent spots against a dark blue background [67]. Since some plant metabolites are not visible under UV light, further derivatization was performed using the *p*-anisaldehyde/sulfuric acid (ASA) reagent. ASA is a widely used reagent for visualizing natural products and produces spots of varying colors based on the compound type and concentration [68]. With the MP2 system, several violet zones concentrated near the solvent front suggested the presence of lipophilic compounds such as triterpenes and phytosterols. A prominent dark blue zone at *hR_F_* 65 can be attributed to monoterpenes or monoterpene alcohols (Figure 3i) [69]. However, using the MP1 developing system, several yellow/orange zones appeared, suggesting the presence of flavonoids such as myricetin, rutin, or quercetin (Figure 3d) [68]. To further confirm the compound classes, derivatization with the NP/PEG reagent was applied. NP/PEG induces fluorescence in phenolic compounds, which can then be visualized under UV 366 nm (Figure 3c). This reagent is particularly effective for detecting flavonoids, isoflavonoids, and phenolic acids [68]. Chromatograms developed using MP 1 displayed numerous orange zones, light blue spots, and red spots at higher *hR_F_* values (Figure 1c). Flavonoids, which appear as yellow spots with ASA, typically fluoresce yellow, orange, or green against a dark blue background with NP/PEG, depending on whether they possess one hydroxyl group (yellow), two hydroxyl groups (orange), or three hydroxyl groups (green) [66]. Light blue zones, on the other hand, can be attributed to phenolic acids which generally appear as light gray spots after ASA derivatization but may be difficult to distinguish against a grayish chromatographic background [67]. Additionally, red spots are commonly associated with chlorophylls [68]. Notably, *H. perforatum* is characterized by the presence of anthraquinone derivatives, hypericin and pseudohypericin. Previous studies on HPTLC profiles of *H. perforatum* extracts have reported that hypericin and pseudohypericin produce red zones under UV 366 nm exposure. Thus, the red-colored spots observed in the HPTLC profiles may correspond not only to chlorophylls but also to these compounds [70,71]. Based on HPLC results, pseudohypericin was identified in the concentrated methanol extract, which can be linked to the observed red spot. However, further targeted analysis is necessary to confirm this observation. In addition, comparisons in the literature suggest that fingerprint profiles under UV 254 nm and 366 nm, prior to derivatization, along with chromatograms obtained after derivatization with ASA and NP/PEG reagent, can indicate the presence of flavonoids. These compounds typically appear as dark spots against a green background under UV 254 nm and as grayish spots under UV 366 nm, as observed for zones at *hR_F_* values of 15, 35, 38, and 53 [67]. The visualization of these zones aligns with literature reports that *H. perforatum* is rich in flavonoids, particularly rutin, hyperoside, quercetin, and isoquercetin, as well as phenolic acids such as caffeic acid, ferulic acid, chlorogenic acid, and gallic acid [72]. This is consistent with the UHPLC analysis results, which confirmed the presence of quercetin, rutin and hyperoside, gallic acid, ferulic acid derivatives, and four isomers of caffeoylquinic acid, in the methanolic *H. perforatum* extract.

### 3.4. HPTLC Antibacterial Activity of Methanolic H. perforatum Extract

Treating infections caused by *S. aureus* and *K. pneumoniae* presents an increasing challenge due to their growing resistance to conventional antibiotics. *S. aureus* is a Gram-positive bacterium known for causing invasive infections, from mild skin and soft tissue infections to more severe conditions such as pneumonia, bacteremia, and osteomyelitis. It has a strong tendency to develop resistance, with methicillin-resistant *S. aureus* (MRSA) being the most well-known variant [73]. Conversely, *K. pneumoniae* is a Gram-negative bacterium responsible for various life-threatening infections, including pneumonia, urinary tract infections, bacteremia, meningitis, and liver abscesses [74]. Both strains are commonly found in the environment, with higher infection risks among vulnerable populations such as newborns, the elderly, and immunocompromised individuals [75]. Given their significant resistance potential, identifying novel compounds with antimicrobial properties is critical to addressing this global threat. The applied MTT bioassay relies on the activity of oxidoreductases in living bacterial cells, which reduce yellow MTT to blue-violet formazan [76]. In the MP1 system, an active compound at *hR_F_* 35 was presumed to belong to the flavonoid class, based on its zone coloration after chemical derivatization (Figure 3e,f). Other flavonoid-related zones exhibited lower antibacterial activity compared to the zone with *hR_F_* 35. Flavonoids are well documented for their antimicrobial activity through diverse mechanisms, including bacterial membrane disruption, inhibition of nucleic acid and protein synthesis, and interference with energy metabolism. Furthermore, these compounds inhibit quorum sensing and biofilm formation, reducing bacterial virulence and growth, which makes them effective broad-spectrum antibacterial agents with potential activity against resistant strains [77]. In the MP2 system, the most prominent zone with antibacterial activity was at *hRf* values of 45 and 60 (Figure 3j,k). Based on the bluish coloration after ASA derivatization, these active compounds are likely monoterpenes. Additionally, an active region from *hR_F_* 85 to the front of the mobile phase was noted, corresponding to phytosterols and triterpene derivatives, as suggested by their coloration with ASA reagent. The stronger antibacterial activity observed in MP 2 highlights the significant contribution of lipophilic components within the extract. Terpenoid compounds, including monoterpenes and triterpenes, exert antibacterial effects by integrating into and disrupting lipid bilayers in microbial cell membranes. Their lipophilic nature increases membrane permeability, disrupting critical cellular processes and ultimately leading to cell death [78]. *H. perforatum* is known to be rich in terpenoids, as confirmed by the literature, which identifies α-pinene as the most abundant monoterpene in this species [79]. Since the present study did not include GC-MS volatile profile examination, it can be speculated that these compounds are responsible for the observed antibacterial activity. Therefore, further analysis of *H. perforatum*’s volatile profile is necessary to confirm this assumption. With additional quantification of antibacterial activity presented in Table 4, the MP2 system stood out as more prominent. This was consistent with the visual inspection of chromatograms, which showed a higher number of active zones with greater intensity compared to MP 1. Furthermore, in both mobile phase applications, the extract demonstrated greater activity against *S. aureus* than *K. pneumoniae*. This observation is expected, as Gram-negative bacteria, such as *K. pneumoniae*, possess a lipophilic outer membrane primarily composed of lipopolysaccharides. This membrane forms a hydrophilic permeability barrier that limits the entry of hydrophobic compounds, thereby reducing their antibacterial efficacy [80]. This structural feature likely explains the lower sensitivity of *K. pneumoniae* to the active compounds in the extract compared to *S. aureus*. Literature data support the stronger antibacterial activity of methanolic *H. perforatum* extracts against Gram-positive bacteria over Gram-negative ones [57]. For instance, in a study by Kakouri et al. [81], *H. perforatum* extract exhibited a minimum inhibitory concentration (MIC) of 0.06 mg/mL and a minimum bactericidal concentration (MBC) of 0.51 mg/mL against *S. aureus*, whereas no activity was observed against tested Gram-negative strains. Furthermore, Avato et al. [82] determined the MICs of *H. perforatum* extracts obtained using methanol, ethyl acetate, chloroform, and petroleum ether against Gram-positive bacterial strains. Their study demonstrated notable activity against three *S. aureus* strains, with the ethyl acetate extract exhibiting MIC values of 12.5 μg/mL, whereas the methanolic extract showed weaker activity, with MIC values of 300 μg/mL. The pronounced antibacterial activity was attributed to the high content of flavonoids, as well as the presence of hyperforins and hypericins. Specifically, hyperforin exhibited an MIC value of 50 μg/mL, while hypericin demonstrated MIC values of 12.5 μg/mL against the tested *S. aureus* strains.

## 4. Materials and Methods

### 4.1. Chemicals and Materials Used for Analyses

The extraction process for *H. perforatum* included water and methanol sourced from Zorka Pharma (Šabac, Serbia). For each assay, several chemicals were used for spectrophotometric analysis. For phytochemical analyses, Folin–Ciocalteu reagent and sodium molybdate were purchased from Sigma-Aldrich (Steinheim, Germany), sodium carbonate from Zorka Pharma (Šabac, Serbia), hydrochloric acid from Zorka Pharma (Šabac, Serbia), and ferric (III) chloride hexahydrate from Loba Chemie Pvt Ltd. (Mumbai, India). Antioxidant property determinations involved ABTS^•+^ solution, DPPH^•^ solution, neocuproine, and potassium molybdate from Sigma-Aldrich (Steinheim, Germany), potassium ferrocyanide from Alkaloid (Skopje, North Macedonia), trichloroacetic acid (TCA) from Superlab (Belgrade, Serbia), and copper (II) chloride and ammonium acetate from Zorka Pharma (Šabac, Serbia). For HPLC analysis, the solvent system and mobile phases were prepared using methanol (Carlo Erba, Val-de-Reuil, France), and acetonitrile (Carlo Erba, Val-de-Reuil, France) with ultrapure water (Smart-DUV instrument, Amtast USA Inc., Lakeland, FL, USA). HPTLC analysis utilized methanol, sodium dihydrogen phosphate, sodium chloride, sodium hydroxide, polyethylene glycol 4000 (PEG 4000), and 10 cm × 10 cm glass HPTLC plates silica gel 60 (Art. 105461), all purchased from Merck (Darmstadt, Germany). Ethyl acetate and toluene were sourced from Zorka Pharma (Šabac, Serbia), while formic acid was obtained from Lach-Ner (Neratovice, Czech Republic). Acetic acid and sulfuric acid were supplied by Centrohem (Stara Pazova, Serbia). Streptomycin, 3-(4,5-dimethylthiazol-2-yl)-2,5-diphenyltetrazolium bromide (MTT), *p*-anisaldehyde and 2-aminoethyl diphenylborinate were from Sigma-Aldrich Chemie GmbH (Steinheim, Germany). Nutrient agar was provided from Lab M (Bury, UK), while Tripton LP0042 and yeast extract LP0021 were obtained from Oxoid LTD (Basingstoke, UK). Luria–Bertani (LB) broth, used for bacterial cultivation, was prepared by dissolving 10 g of tryptone, 5 g of yeast extract, and 5 g of sodium chloride in 1 L of distilled water, followed by autoclaving at 121 °C.

The standards used for the confirmation of phenolic compounds derived from *H.perforatum* extracts were as follows: 3,4-dihydroxybenzoic acid (Protocatehuic acid) (Fluka, >99% purity, Charlotte, North Carolina, USA); Gallic acid (Chem Faces, >98% purity, Wuhan, Hubei, China); Chlorogenic acid (Chem Faces, >98% purity, Wuhan, Hubei, China); Rosmarinic acid (Chem Faces, >98% purity, Wuhan, Hubei, China); Catechin (Chem Faces, >98% purity, Wuhan, Hubei, China); Epicatechin (Chem Faces, >98% purity, Wuhan, Hubei, China); Procyanidin B2 (Chem Faces, >98% purity, Wuhan, Hubei, China); Kaempferol (Chem Faces, >98% purity, Wuhan, Hubei, China); Quercetin (Chem Faces, >98% purity, Wuhan, Hubei, China); Myricetin (Chem Faces, >98% purity, Wuhan, Hubei, China); Quercetin 3-*O*-rhamnoside (Quercitrin) (Chem Faces, >98% purity, Wuhan, Hubei, China); Quercetin 3-*O*-glucoside (Hyperoside) (Extrasynthese, >99% purity, Lyon, France); Myricetin 3-*O*-glucoside (Extrasynthese, >99% purity, Lyon, France); Quercetin 3-*O*-(6”-*O*-acetyl)-beta-D-glucopyranoside (Extrasynthese, >95% purity, Lyon, France); Rutin (Chem Faces, >98% purity); Naringenin (Chem Faces, >98% purity, Wuhan, Hubei, China).

### 4.2. Plant Material and Extraction Protocol for Spectrophotometric Analysis

The plant material used in this study was sourced from *Adonis d.o.o.*, a tea manufacturer based in Sokobanja (Serbia). For spectrophotometric analysis, the extraction process for aqueous extracts was designed to replicate standard tea preparation, following the instructions provided on the packaging. The infusion was prepared using boiling water as the solvent, with a plant material-to-water ratio of 1:50 (resulting in an aqueous tea infusion—ATI). Additionally, a methanolic extract (MW) was prepared using 80% methanol, maintaining the same ratio. Aqueous tea infusion (ATI) and methanolic extract (MW) were used for spectrophotometric analysis and/or UHPTLC bioautography. Furthermore, a pure methanol (M) extract was prepared and included in UHPLC chromatographic analysis in order to extract and detect less polar compounds present in *H. perforatum*.

For preparation of the M extract of *H. herba,* plant material was extracted using 100% methanol (1:10 *w/v*), on a mechanical shaker, for 1 h. The resulting mixture was then centrifuged for 10 min at 4000× *g*, and the obtained supernatant was evaporated to dryness using a rotary evaporator (Heidolph, Laborota 4000, Schwabach, Germany) under reduced pressure at 30 °C. The residue after evaporation was dissolved in 1 mL of 100% methanol. Before chromatographic analysis, all extracts (ATI, MW, and M) were filtered through a 0.22 mm filter. Additionally, the ATI was further purified by filtration through cartridges. Elution of extracted compounds from cartridges was achieved using pure methanol.

### 4.3. Spectrophotometric Determination of Proximate Phytochemical Composition and Antioxidant Activity

The proximate phytochemical composition for both solvent extracts was evaluated using various assays, including the determination of total phenolic content (TPC) and total content of hydroxycinnamic acid derivatives (HCAs). Antioxidant properties were also assessed using methods such as in vitro phosphomolybdenum total antioxidant capacity (TAC), ferric reducing power (FRP), cupric reducing antioxidant capacity (CUPRAC), 2,2-diphenyl-1-picrylhydrazyl radical (DPPH^•^) quenching ability, and ABTS^⦁+^ assays. These analyses were conducted following the methodology outlined by Kilibarda et al. [83]. Additionally, the total tannin content was determined following the methodology described by Vijayalaxmi et al. [84]. All experiments were performed in triplicate (n = 3) using both methanolic and aqueous extracts, with measurements conducted on a spectrophotometer (UV-1800 model, Shimadzu, USA Manufacturing Inc., Canby, Oregon, USA). The results are expressed based on the dry weight (DW) of the plant material.

### 4.4. UHPLC Q-ToF MS Analysis of Bioactive Compounds

The separation and characterization of bioactive compounds in *H. herba* extracts were performed using an Agilent 1290 Infinity ultra-high-performance liquid chromatography (UHPLC) system coupled with a quadrupole time-of-flight mass spectrometry (6530C Q-ToF-MS) (Agilent Technologies, Inc., Santa Clara, CA, USA). The applied UHPLC method and Q-ToF operating parameters were previously reported in detail by Kostić et al. [85]. Data-dependent acquisition (DDA) was employed for suspect screening, using the Auto MS/MS acquisition mode (100–1700 *m/z*, scan rate 1 spectra/s). The applied collision energy was fixed and set at 30 eV. Agilent MassHunter software was used for instrument control, data collections (monoisotopic mass of all precursor ions and MS/MS fragments-product ions), and analysis. Phenolic compounds, naphthodianthrones, xanthones, and coumestans were confirmed in negative ionization mode, whereas polycyclic polyprenylated acylphloroglucinols (PPAPs) were analyzed in positive ionization mode. All bioactive compounds were putatively identified based on monoisotopic mass and MS fragmentation and confirmed through comparison with available standards (for some phenolic compounds) or literature data. Phenolic compounds confirmed by available standards are labeled in Table 3. The fragmentation patterns (MS/MS spectra) of these standards are presented in the Supplementary Material S5. Accurate masses of components were calculated using ChemDraw software (version 12.0, CambridgeSoft, Cambridge, MA, USA) and ChemCalc software (https://www.chemcalc.org/) (accessed on 10 February 2025). Additionally, compound identification was supported by searches in CAS SciFinder-n (https://scifindern.cas.org/, accessed on 10 February 2025) and PubChem (https://pubchem.ncbi.nlm.nih.gov/, accessed on 10 February 2025) databases based on molecular formulas and structures.

### 4.5. HPTLC Analysis

Due to the high diversity of bioactive compounds identified by UHPLC Q-ToF MS, the MW extract of *Hyperici herba* was selected for HPTLC analysis and bioautography testing. A methanolic extract (20 μL) of *H. perforatum* was applied as 8 mm bands onto HPTLC glass silica gel plates using a Linomat 5 (CAMAG, Muttenz, Switzerland). The bands were applied starting from the lower edge (8 mm) and with a minimum spacing of 10 mm from each side. Plates were developed to a distance of 70 mm in a saturated Twin Trough Chamber 10 cm × 10 cm (CAMAG), with a mobile phase composed of toluene/ethyl acetate/methanol (55/40/5, *v/v/v*) and ethyl acetate/toluene/formic acid/water (16/4/3/2, *v/v/v/v*). After development, the plates were dried in a stream of cold air for 5 min. Digital plate images were taken using a smartphone (iPhone 15 Pro, Apple Inc., Cupertino, CA, USA), equipped with a 48-megapixel camera, and stored in JPEG format for further processing.

To evaluate the content of natural products, derivatization was performed using *p*-anisaldehyde-sulfuric acid (ASA) and 2-aminoethyldiphenylborinate (NEU) reagents with a Chromatogram Immersion device (CAMAG). The ASA reagent was prepared by mixing 1.5 mL of *p*-anisaldehyde with 200 mL methanol, 25 mL glacial acetic acid, and 12.5 mL of concentrated sulfuric acid. After derivatization, the plates were heated to 110 °C for 10 min, allowing spot visualization under white light. The natural product (NP) reagent was prepared by dissolving 0.5 g of 2-aminoethyldiphenylborinate in 100 mL of methanol. After development, the chromatograms were subjected to a sequential immersion process: first with the NP solution, followed by drying with a stream of hot air, and then in a 5% (*w*/*v*) polyethylene glycol 4000 (PEG) solution in methanol. Plates were evaluated under UV 366 nm.

### 4.6. Antibacterial Assays

The antibacterial activity against *S. aureus* ATCC 6538 and *K. pneumoniae* ATCC 29,665 was evaluated using HPTLC–direct bioautography. Two LB aliquots of 10 mL were inoculated with a single bacterial colony, taken from previously cultivated strains on nutrient agar for 24 h at 37 °C. The inoculated broths were then incubated for 18 h in an orbital Shaker–Incubator ES-20 (BioSan, Riga, Latvia) at 37 °C and 220 rpm. The bacterial growth was monitored by measuring the optical density (OD) at 600 nm. Suspensions for derivatization were prepared by inoculating 200 mL of LB medium in 500 mL flasks with 0.2 mL of overnight bacterial cultures (incubated for 18 h). The flasks were incubated at 37 °C in the shaker until the cultures reached the exponential growth phase (OD_600_ = 0.6). The developed plates were then briefly immersed in these bacterial suspensions and incubated in a humidity chamber at 37 °C under aerobic conditions for 90 min to allow bacterial growth on the plate surface. Antibacterial zones were visualized using a 0.1% solution of MTT in a phosphate buffer (0.1 mol/L, pH 7.2). An additional one-hour incubation was performed, and a positive reaction, indicated by a color change, was noted. Streptomycin was used as a reference standard for expressing antibacterial activity.

### 4.7. Image Processing and Data Acquisition

Digital images of bioautograms, containing both the extract and the corresponding standard, were processed using ImageJ software 2.16.0. (https://imagej.net/downloads, accessed 23 January 2025), following a modified version of the method described by Chen et al. [86]. Prior to processing, all plate images were standardized to uniform dimensions and converted to binary format (Selection/Image/Type/8-bit). Background noise reduction was performed using the “Subtract background” option (Process/Subtract Background/Rolling Ball Radius: 1000 pixels). The first band was outlined using the “Rectangular selection tool”. All subsequent bands were selected (Analyze/Gels/Select Next Lane), and plot profiles were generated (Analyze/Gels/Plot Lane), illustrating pixel intensity as a function of distance. The *x*-axis represented distance along the chromatographic line, while the *y*-axis depicted pixel intensity. Peak areas corresponding to separated zones on the chromatograms were measured using the “Wand tool”. The obtained peak areas in pixels were used to construct a calibration curve for streptomycin. The calibration curve was generated by plotting peak areas against streptomycin concentrations expressed in μg/band. Linear ranges were determined through least squares regression analysis. Furthermore, the activity of each extract was quantified as streptomycin equivalents (StrpE).

### 4.8. Statistical Analysis

Statistical analysis was conducted using R Studio 4.3.1 software, employing analysis of variance (ANOVA) and Tukey’s post hoc test to determine significant differences among solvents (considered statistically significant at *p* < 0.05).

## Figures and Tables

**Table 1:** Proximate phytochemical composition of *H. perforatum* methanolic extract (MW) and infusion tea (aqueous extract, ATI).

Analysis	Infusion Tea (ATI)	Methanolic Extract (MW)
TPC (mg/g GAE DW) *	26.48 ± 0.96 ^b^	31.38 ± 0.52 ^a^
HCAs (mg/g CGAE DW)	3.28 ± 0.24 ^a^	4.22 ± 0.20 ^a^
Total tannin content (mg/g TAE DW)	12.83 ± 2.50 ^b^	7.51 ± 2.72 ^a^

* DW—dry weight; TPC—total phenolic content; HCA—total dyhydroxicinnamic acid derivative content; GAE—gallic acid equivalent; CGAE—chlorogenic acid equivalent; TAE—tannic acid equivalent. Values are the means of three different measurements (*n* = 3) ± standard deviation; different letters (a,b) in the same row indicate significant differences among extraction solvents within the assay at *p* < 0.05.

**Table 2:** Identification and characterization of phenolic compounds of *H. perforatum* infusion tea and methanolic extract, using UHPLC Q-ToF MS (untarget analysis). Identified compounds, retention time (RT), molecular formula, calculated and exact mass, and MS fragments are presented in the table.

No.	RT	Compounds	Formulas	Calculated Mass	*m/z* Exact Mass	mDa	MS Fragments (Main Fragment)	**Extracts**	
								MW	ATI
Hydroxybenzoic acid and derivatives									
**1**	4.33	**Hydroxybenzoic acid**	C_7_H_5_O_3_^−^	137.0239	137.0242	0.33	**108.0205(100)**	**+**	**+**
**2**	2.37	Dihydroxybenzoic acid (Protocatehuic acid)*	C_7_H_5_O_4_^−^	153.0188	153.0187	−0.08	**108.0206(100)**, 109.0281	**+**	**+**
**3**	2.02	Gallic acid *	C_7_H_5_O_5_^−^	169.0137	169.0132	−0.5	**107.0127(100)**, 151.0019, **125.0224**	**+**	**+**
**4**	1.84	**Dihydroxybenzoic acid hexoside is. I**	C_13_H_15_O_9_^−^	315.0716	315.0716	−0.01	**108.0205(100)**, 152.0100, **109.0276**	**+**	**−**
**5**	2.44	**Dihydroxybenzoic acid hexoside is. II**	C_13_H_15_O_9_^−^	315.0716	315.0716	−0.01	**108.0206(100)**, 152.0103, **109.0274**, 153.0168	**+**	**+**
**6**	4.12	**Dihydroxybenzoic acid hexoside is. III**	C_13_H_15_O_9_^−^	315.0716	315.0717	0.09	**109.0283(100)**, **153.0181**, 152.0099, 108.0204	**−**	**+**
**7**	3.10	**Vanillic acid hexoside**	C_14_H_17_O_9_^−^	329.0873	329.0876	0.34	**108.0207(100)**, 152.0103, **123.0437**, **167.0336**	**+**	**+**
**8**	1.81	**Gallic acid hexoside is. I**	C_13_H_15_O_10_^−^	331.0665	331.0662	−0.32	**168.0053(100)**, 125.0233, 149.9945, 124.0151, 313.0544	**−**	**+**
**9**	2.85	**Gallic acid hexoside is. II**	C_13_H_15_O_10_^−^	331.0665	331.0662	−0.32	**125.0232(100)**, 169.0125, 124.0151, **168.0059**	**−**	**+**
**10**	5.21	**Syringic acid hexoside**	C_15_H_19_O_10_^−^	359.0978	359.0985	0.68	**138.0309(100)**, 182.0204, 123.0072, 153.0539, 166.9970, **197.0446**	**+**	**−**
Hydroxycinnamic acid derivatives									
**11**	6.44	**Coumaric acid hexoside**	C_15_H_17_O_8_^−^	325.0923	325.0911	−1.24	**119.0485(100)**, **163.0385**, 145.0286	**−**	**+**
**12**	5.87	***p*** **-coumaroylquinic acid is. I**	C_16_H_17_O_8_^−^	337.0923	337.0927	0.36	**119.0488(100)**, **163.0394**, **191.0551**, 155.0337, 173.0443	**+**	**+**
**13**	7.06	***p*** **-coumaroylquinic acid is. II**	C_16_H_17_O_8_^−^	337.0923	337.0927	0.36	**173.0441(100)**, 119.0495, **163.0386**, **191.0507**, 155.0374, 127.037	**+**	**+**
**14**	5.58	**Caffeic acid hexoside**	C_15_H_17_O_9_^−^	341.0873	341.0884	1.14	**135.0436(100)**, **179.0337**, 161.0234	**+**	**−**
**15**	3.71	**Caffeoylquinic acid is. I**	C_16_H_17_O_9_^−^	353.0873	353.0873	0.04	**191.0546(100)**, 135.0442, **179.0341**, 161.0231, 173.0443	**+**	**+**
**16**	4.79	**Caffeoylquinic acid is. II**	C_16_H_17_O_9_^−^	353.0873	353.0873	0.04	**191.0546(100)**, 135.0434, **179.0344**, 161.0234, 173.0441, 127.039	**+**	**+**
**17**	6.41	Caffeoylquinic acid is. III (Chlorogenic acid)*	C_16_H_17_O_9_^−^	353.0873	353.0873	0.04	**191.0545(100)**, 135.0444, 173.0448, **179.0342**, 161.023, 127.0388	**+**	**+**
**18**	6.93	**Caffeoylquinic acid is. IV**	C_16_H_17_O_9_^−^	353.0873	353.0873	0.04	**191.0546(100)**, 135.0438, 161.0249, 173.0446, **179.0351**, 127.0424	**+**	**−**
**19**	8.67	Rosmarinic acid *	C_18_H_15_O_8_^−^	359.0767	359.078	1.31	**161.0230(100)**, 135.044, **179.0333**, 123.0452, **197.0429**	**−**	**+**
**20**	6.46	**Feruloylquinic acid**	C_17_H_19_O_9_^−^	367.1029	367.1039	0.99	**134.0364(100)**, **193.0494**, **191.0536**, 149.0593, 155.0335, 173.0443	**+**	**−**
**21**	4.00	**Coumaroylquinic acid hexoside**	C_22_H_27_O_13_^−^	499.1452	499.1443	−0.87	**163.0386(100)**, 119.0488, 173.0432, 155.0331	**−**	**+**
**22**	8.55	**Dicaffeoylquinic acid**	C_25_H_23_O_12_^−^	515.119	515.119	0.05	**173.0448(100)**, **179.0338**, 191.0547, **353.0858**, 135.0435, 161.0250, 155.033, 209.0774	**+**	**−**
**23**	5.48	**Caffeoylquinic acid hexoside**	C_22_H_27_O_14_^−^	515.1401	515.1386	−1.48	**179.0334(100)**, **191.0542**, **341.0845**, **135.0436**, 515.1403, 323.0764, **353.0866**, 161.0238, 155.0320, 173.0445	**−**	**+**
Flavan-3-ols and procyanidins									
**24**	6.22	Catechin *	C_15_H_13_O_6_^−^	289.0712	289.0708	−0.41	**123.044(100)**, 109.0283, 125.0235, 151.0388, 137.0232, 203.0701, 149.0246, 161.0584, 221.0802, 245.0814	**+**	**−**
**25**	6.98	Epicatechin *	C_15_H_13_O_6_^−^	289.0712	289.0708	−0.41	**123.0440(100)**, 109.0286, 125.0235, 151.039, 121.0285, 137.0233, 203.0703, 149.0242, 161.0583, 221.0810, 245.0798	**+**	**−**
**26**	6.81	Procyanidin B-type dimer (Procyanidin B2) *	C_30_H_25_O_12_^−^	577.1346	577.1337	−0.9	**289.0702(100)**, **407.0761**, 125.0233, 245.0781, 161.024, 137.023, **273.0403**, 205.0472, **425.0861**, **451.1014**, 109.0277, 179.0334	**+**	**+**
Flavonol aglycones and glycosides									
**27**	10.37	Kaempferol *	C_15_H_9_O_6_^−^	285.0399	285.0395	−0.41	**285.0390(100)**, 185.058, 187.0391, 239.0339, 229.0476, 159.0432, 211.0389, 143.0504, 257.0286, 151.0019, 267.0296	**+**	**−**
**28**	9.83	**Dehydroquercetin**	C_15_H_7_O_7_^−^	299.0192	299.0203	1.12	**151.0026(100)**, 121.0284, 107.0127, 271.0236, 299.0173, **178.9966**, 227.034, 243.0274	**+**	**−**
**29**	9.64	Quercetin *	C_15_H_9_O_7_^−^	301.0348	301.0356	0.77	**151.0028(100)**, 121.0284, 107.0129, **178.9974**, 149.0233, 245.0438, 229.0490, 273.0379, 301.0339	**+**	**+**
**30**	7.20	Myricetin *	C_15_H_9_O_8_^−^	317.0297	317.031	1.26	**109.0287(100)**, 151.0034, 243.1227, 163.0029, 125.0218, 179.0043, 107.0122, 227.0327, 257.1385, 271.0259	**+**	**−**
**31**	7.54	**Dihydromyricetin**	C_15_H_11_O_8_^−^	319.0454	319.0448	−0.59	**139.0386(100)**, 109.0291, 183.0273, 153.0196, 258.0154, 165.0193, 201.0100, 214.0265, 242.0161	**+**	**−**
**32**	8.19	**Quercetin 3-*O*-pentoside (Guaijaverin)**	C_20_H_17_O_11_^−^	433.0771	433.0775	0.41	**300.0264(100**), 301.0313, 271.0238, 255.0285, **151.0024**, **179.0004**	**+**	**−**
**33**	8.30	Quercetin 3-*O*-rhamnoside (Quercitrin) *	C_21_H_19_O_11_^−^	447.0927	447.0933	0.56	**300.0263(100)**, 301.033, 271.024, **151.0045**, 255.0288, 178.9976, 243.0286, 227.0340, 285.0388	**+**	**+**
**34**	7.92	Quercetin 3-O-hexoside (Hyperoside) *	C_21_H_19_O_12_^−^	463.0877	463.0879	0.25	**300.0261(100)**, 301.0313, 271.0237, 255.0284, **151.0027**, **178.9973**	**+**	**+**
**35**	7.94	**Quercetin 3-*O*-glucuronide (Miquelianin)**	C_21_H_17_O_13_^−^	477.0669	477.067	0.08	**301.0347(100)**, **151.0022**, **178.9974**, 273.0391, 255.0285, 229.049	**+**	**+**
**36**	7.45	Myricetin 3-*O*-hexoside *	C_21_H_19_O_13_^−^	479.0826	479.084	1.43	**316.0196(100)**, 317.0264, 271.0237, 479.0813, 287.0178, 257.0445, **178.9988**, **151.0022**	**+**	**−**
**37**	8.26	**Quercetin 3-*O*-(6”-*O*-acetyl)hexoside**	C_23_H_21_O_13_^−^	505.0982	505.0988	0.58	**300.0266(100)**, 301.0308, 271.0233, 255.0288, 243.0281, **151.0007**	**+**	**+**
**38**	8.80	Quercetin 3-*O*-(6”-*O*-acetyl)-beta-D-glucopyranoside *	C_23_H_21_O_13_^−^	505.0982	505.0988	0.58	**300.0269(100)**, 301.0304, 271.0233, 255.0284, 243.0284, **151.0021**	**+**	**+**
**39**	7.79	Quercetin 3-*O*-(6”-rhamnosyl)hexoside (Rutin)*	C_27_H_29_O_16_^−^	609.1456	609.1462	0.64	**300.0264(100)**, 609.1442, 301.0327, 271.024, **151.003**, **178.9977**, 255.0303, 243.0288	**+**	**+**
Other flavonoids									
**40**	10.16	Naringenin *	C_15_H_11_O_5_^−^	271.0606	271.0611	0.45	**119.0483(100)**, 151.0036, 107.0124, 187.037, 145.0273	**+**	**−**
**41**	12.88	**Trimethoxyflavone (like Salvigenin)**	C_18_H_15_O_6_^−^	327.0869	327.0869	0.04	**327.0866(100)**, 297.0396, 328.0895, 311.0548, 283.0241, **312.0599**, **298.0422**, 271.0253	**+**	**−**
**42**	10.69	**I3,II8-Biapigenin**	C_30_H_17_O_10_^−^	537.0822	537.0826	0.43	**151.0028(100)**, **385.0707**, **443.0397**, 537.0821, 107.0129, 417.0594, 493.0917, 267.0285, 451.0813, 375.0490, 309.0395	**+**	**−**

**Abbreviations:** “is”—isomers; MW—methanol/water (80/20 *v/v*) extract; ATI—aqueous tea infusion; “−”—non-identified compounds; “+”—identified compounds; *—compounds confirmed by available standards.

**Table 3:** Putative identification of naphthodianthrones, polycyclic polyprenylated acylphloroglucinols (PPAPs), xanthones, and other biocompounds derived from *H. Perforatum* by UHPLC Q-ToF MS. Identified compounds, retention time (RT), molecular formula, calculated and exact mass, and MS fragments are presented in the table.

No.	RT	Tentatively Identified Compounds	Formulas	Calculated Mass	*m/z* Exact Mass	mDa	MS Fragments (Main Fragment)	Extracts			Previously Reported in *Hypericum*
								ATI	MW	M	
***Naphthodianthrones***											
**43**	16.65	**Pseudohypericin**	C_30_H_15_O_9_^−^	519.0716	519.0734	1.79	**519.0746(100)**, 520.0773, 487.0466, 503.044, 475.0752, 449.0711, 443.0575, 421.069	**−**	**−**	**+**	[14,15,16,17]
**44**	15.90	**Pseudoprotohypricin**	C_30_H_17_O_9_^−^	521.0873	521.0891	1.84	**521.0905(100)**, 522.0924, 477.0988, 423.0885, 379.0945, 449.1025	**−**	**−**	**+**	[14,15,16,17]
***Polycyclic polyprenylated acylphloroglucinols*** **(PPAPs)**											
**45**	14.69	**Hyperfirin**	C_30_H_45_O_4_^+^	469.3318	469.3322	0.42	**401.2703(100)**, 469.3346, 345.2076, 413.2704, 223.0977, 333.2072, 279.1601, 277.1451, 291.1602, 305.1757, 319.1912, 357.2073	**−**	**+**	**+**	[11,18,19]
**46**	14.93	**Adhyperfirin**	C_31_H_47_O_4_^+^	483.3474	483.3484	0.97	**415.2856(100)**, 483.3488, 427.2858, 359.2228, 293.1737, 237.1139, 371.2231, 347.2227	**−**	**−**	**+**	[11,18,19]
***Furano-polycyclic polyprenylated acylphloroglucinols*** **(FPPAPs)**											
**47**	13.13	**FPPAP derivative 1 (like Hyperformitin J, K, L or M)**	C_30_H_45_O_5_^+^	485.3267	485.3284	1.7	**485.33(100)**, 467.3182, 399.2547, 411.2547, 385.2388, 333.2074, 331.1917	**−**	**−**	**+**	[20]
**48**	15.54	**FPPAP derivative 2 (like Hyperioxide D)**	C_35_H_51_O_6_^+^	567.3686	567.3712	2.64	**293.1401(100)**, 275.1303, 331.1914, 329.1794, 347.1868, **349.1993**, 443.2847, 425.2754, 481.3098, 499.3120, 549.3530	**−**	**−**	**+**	[21]
**49**	16.49	**FPPAP derivative 3 (like Hyperformitin C, Hyperformitin D (Type A PPAPs) or Hyperfol F (Type B PPAPs))**	C_35_H_53_O_6_^+^	569.3842	569.3876	3.39	**293.1398(100)**, **347.1865**, **365.1978**, 275.1314, 331.1926, 329.1799, 499.3407, 483.3136	**−**	**−**	**+**	[20,22]
**50**	13.75	**FPPAP derivative 4 (unknown)**	C_35_H_53_O_7_^+^	585.3791	585.3819	2.77	**293.1396(100)**, 275.1304, **347.1863**, **365.1996**, 329.1782, 481.2988, 517.3159, 567.3643	**−**	**+**	**+**	/
**51**	15.46	**FPPAP derivative 5 (unknown)**	C_35_H_53_O_7_^+^	585.3791	585.3819	2.77	**293.1395(100)**, 275.1302, **347.1877**, 329.1783, **365.2011**, 567.3697, 549.3622, 517.3203, 499.3137, 481.3050	**−**	**−**	**+**	/
**52**	15.15	**FPPAP derivative 6 (like Hyperidione F)**	C_35_H_55_O_7_^+^	587.3948	587.3985	3.72	**293.1397(100)**, 294.1433, 275.1301, **349.2019**, 331.1920, 569.3860, 277.1448, 551.3745, 221.0827	**−**	**+**	**+**	[23,24]
**53**	14.5	**FPPAP derivative 7 (unknown)**	C_35_H_55_O_8_^+^	603.3897	603.3928	3.11	**293.1399(100)**, **347.2873**, **365.1983**, 329.1781, 275.1300, 441.2648, 481.3007, 499.3105, 567.3702, 585.3797	**−**	**+**	**+**	/
**54**	13.89	**FPPAP derivative 8 (unknown)**	C_35_H_55_O_9_^+^	619.3846	619.387	2.39	**293.1395(100)**, 275.1302, **347.1876**, **365.1980**, 499.3079, 511.3095, 529.3184, 565.3538, 583.3640, 601.3745	**−**	**+**	**+**	/
***Xanthones***											
**55**	8.98	**Tetrahydroxyxanthone (like Norathyriol)**	C_13_H_7_O_6_^−^	259.0243	259.0242	−0.06	**259.0236(100)**, 109.0285, 215.0336, 187.0388, 159.0437, 231.028, 151.0022	**−**	**+**	**+**	[17,25]
**56**	12.87	**(2 or 8) Prenyl-tetrahydroxyxanthone**	C_18_H_15_O_6_^−^	327.0869	327.0869	0.04	**327.0866(100)**, 297.0396, 328.0895, 311.0548, 283.0241, **258.0147**, 271.0253	**−**	**+**	**−**	[17,25]
**57**	13.60	**γ-mangostin**	C_23_H_23_O_6_^−^	395.1495	395.1525	3.04	**272.0303(100)**, 271.0237, 283.0234, 326.0773, 395.1488, 258.0179, 243.0297	**−**	**+**	**−**	[17,25]
**58**	7.38	**Mangiferin**	C_19_H_17_O_11_^−^	421.0771	421.0773	0.21	**258.0153(100)**, 259.0206, 301.0362, 331.0448, 271.0235	**−**	**+**	**+**	[25,26,27]
***Other compounds (Coumestan)***											
**59**	10.08	**Wedelolactone**	C_16_H_9_O_7_^−^	313.0348	313.0359	1.07	**269.0441(100)**, 225.0543, 241.049, 270.0471, 197.0596, 181.0658, 210.0320, 133.0266	**−**	**+**	**+**	[28]

**Abbreviations:** “is.”—isomers; MW—methanol/water (80/20 *v/v*) extract (methanolic extract); M—methanol extract; ATI—aqueous tea infusion; “−”—non-identified compounds; “+”—identified compounds.

**Table 4:** Antioxidant activity of *H. perforatum* methanolic extract (MW) and infusion tea (aqueous extract, ATI).

Analysis	Infusion Tea (ATI)	Methanolic Extract (MW)
ABTS^⦁+^ (μmol Trolox/g DW) *	176.48 ± 2.32 ^a^	130.49 ± 1.89 ^b^
DPPH^⦁^ (μmol Trolox/g DW)	132.96 ± 0.96 ^b^	149.99 ± 1.31 ^a^
TAC (mg/g AAE DW)	20.73 ± 2.42 ^b^	32.31 ± 0.50 ^a^
FRP (mg/g AAE DW)	21.08 ± 0.71 ^b^	30.58 ± 3.01 ^a^
CUPRAC (mg/g AAE DW)	27.50 ± 1.82 ^a^	20.06 ± 2.57 ^b^

* ABTS^⦁+^—2,2′-Azino-bis(3-ethylbenzothiazoline-6-sulfonic acid) radical cation; DPPH^⦁^—2,2-diphenylpicrylhydrazyl radical; TAC—total antioxidant capacity determined via in vitro phosphomolybdenum assay; FRP—ferric reducing power; CUPRAC—cupric reducing antioxidant capacity; Trolox- 6-hydroxy-2,5,7,8-tetramethylchroman-2-carboxylic acid; AAE—ascorbic acid equivalent. Values are the means of three different measurements (*n* = 3) ± standard deviation; different letters (a,b) in the same row indicate significant differences among extraction solvents within the assay at *p* < 0.05.

**Table 5:** Antibacterial activity of *H. perforatum* extract expressed as streptomycin equivalents (mean value for triplicate ± standard deviation) per mL of extract for the chromatograms obtained with two mobile phases.

	*S. aureus* Assay	*K. pneumoniae* Assay
	StrpE (mg/mL)	StrpE (mg/mL)
MP 1	7.16 ± 0.52	5.13 ± 0.30
MP 2	12.35 ± 0.96	9.70 ± 0.67

## Data Availability

Data will be made available on request.

## Supplementary Materials

The following supporting information can be downloaded at https://www.mdpi.com/article/10.3390/plants14091377/s1. Figure S1: MS base peak chromatograms of (a) aqueous tea infusion (ATI); (b) methanol/water (80/20 *v*/*v*) extract (MW); and (c) methanol extract (M), in positive ionization modes; (d) aqueous tea infusion (ATI); (e) methanol/water (80/20 *v*/*v*) extract (MW); and (f) methanol extract (M), in negative ionization modes; for peak annotation, see retention times in Table 2 and Table 3; Figure S2: Base peak chromatograms of MS/MS product ions of (a) aqueous tea infusion (ATI); (b) methanol/water (80/20 *v*/*v*) extract (MW); and (c) methanol extract (M), in positive ionization modes; (d) aqueous tea infusion (ATI); (e) methanol/water (80/20 *v*/*v*) extract (MW); and (f) methanol extract (M), in negative ionization modes; Figure S3: Characteristic MS/MS fragmentation pattern (collision-induced dissociation (CID) mass spectra), with major fragments of pseudohypericin (Agilent, Q-ToF, ESI(–), CE = 30 eV). The circled mass indicates the major fragment obtained by MS/MS fragmentation of this compound; Figure S4: Characteristic MS/MS fragmentation patterns (collision-induced dissociation (CID) mass spectra), with major fragments of (a) hyperfirin; (b) FPPAP derivative 3 (compound like Hyperformitin C or Hyperformitin D); (c) FPPAP derivative 6 (compound like Hyperidione F), (Agilent, Q-ToF, ESI(+), CE = 30 eV). Proposed fragmentation pathways of these tentatively identified compounds are presented in Figure 5 (manuscript); Figure S5: Fragmentation patterns (MS/MS spectra) of phenolic standards, which are used for confirmation of phenolic compounds found in *H. perforatum* extracts. Compounds: (2) 3,4-dihydroxybenzoic acid (Protocatehuic acid) (Fluka, >99% purity, Charlotte, North Carolina, USA); (3) Gallic acid (Chem Faces, >98% purity, Wuhan, Hubei, China); (17) Chlorogenic acid (Chem Faces, >98% purity, Wuhan, Hubei, China); (19) Rosmarinic acid (Chem Faces, >98% purity, Wuhan, Hubei, China); (24) Catechin (Chem Faces, >98% purity, Wuhan, Hubei, China); (25) Epicatechin (Chem Faces, >98% purity, Wuhan, Hubei, China); (26) Procyanidin B2 (Chem Faces, >98% purity, Wuhan, Hubei, China); (27) Kaempferol (Chem Faces, >98% purity, Wuhan, Hubei, China); (29) Quercetin (Chem Faces, >98% purity, Wuhan, Hubei, China); (30) Myricetin (Chem Faces, >98% purity, Wuhan, Hubei, China); (33) Quercetin 3-*O*-rhamnoside (Quercitrin) (Chem Faces, >98% purity, Wuhan, Hubei, China); (34) Quercetin 3-*O*-glucoside (Hyperoside) (Extrasynthese, >99% purity, Lyon, France); (36) Myricetin 3-*O*-glucoside (Extrasynthese, >99% purity, Lyon, France); (37/38) Quercetin 3-*O*-(6”-*O*-acetyl)-beta-D-glucopyranoside (Extrasynthese, >95% purity, Lyon, France); (39) Rutin (Chem Faces, >98% purity, Wuhan, Hubei, China); (40) Naringenin (Chem Faces, >98% purity, Wuhan, Hubei, China).

## Abbreviations

The following abbreviations are used in this manuscript:

DW	Dry weight
TPC	Total phenolic content
HCAs	Total dyhydroxicinnamic acid derivative content
GAE	Gallic acid
CGAE	Chlorogenic acid equivalent
TAE	Tannic acid equivalent
ABTS^⦁+^	2,2′-Azino-bis (3-ethylbenzothiazoline-6-sulfonic acid) radical cation
DPPH^⦁^	2,2-diphenylpicrylhydrazyl radical
TAC	Total antioxidant capacity determined via in vitro phosphomolybdenum assay
FRP	Ferric reducing power
CUPRAC	Cupric reducing antioxidant capacity
AAE	Ascorbic acid equivalent
StrpE	Streptomycin equivalent

## Appendix A

**Table A1:** Regression data.

Bacterial Strain	Regression Equation	R^2^	Linear Range (μg)	LOD (μg)	LOQ (μg)
*K. pneumoniae*	y = 406141x + 3222862	0.993	10–45	3.8	11.4
*S. aureus*	y = 623224x − 650809	0.994	10–45	3.4	10.2
