# Supplementary material for: Phytochemical Profile and Biological Activities of Rtanj’s Hypericum perforatum Infusion Tea and Methanolic Extracts: Insights from LC-MS/MS and HPTLC–Bioautography
Source: Plants (Basel). 2025 May 1;14(9):1377. doi: 10.3390/plants14091377 (PMC12073223; doi:10.3390/plants14091377)
Supplement: Supplementary file 1 [file plants-14-01377-s001.zip › plants-3580661-supplementary.pdf]

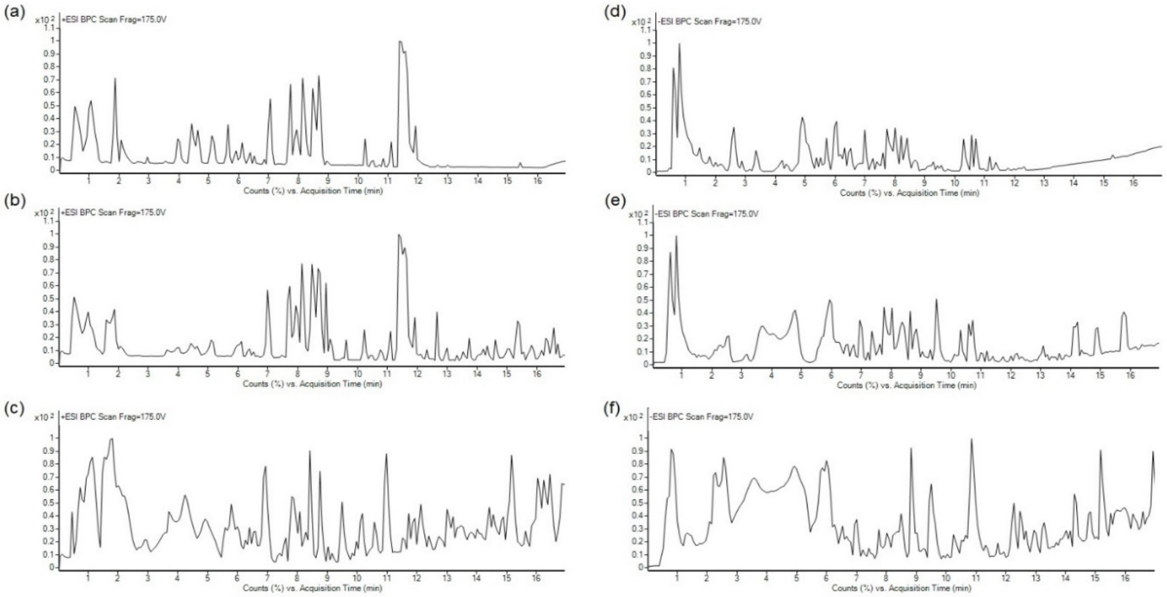

Figure S1. MS-Base peak chromatograms of: **(a)** Aqueous tea infusion (ATI); **(b)** Methanol/water (80/20 *v*/*v*) extract (MW); and **(c)** Methanol extract (M), in **positive** ionization modes; **(d)** Aqueous tea infusion (ATI); **(e)** Methanol/water (80/20 *v*/*v*) extract (MW); and **(f)** methanol extract (M), in **negative** ionization modes; for peak annotation see retention times in Table 2 and Table 3. The peaks of all identified compounds were extracted from these chromatographs based on monoisotopic masses of their precursor ions (Table 2 and Table 3 (manuscript)).

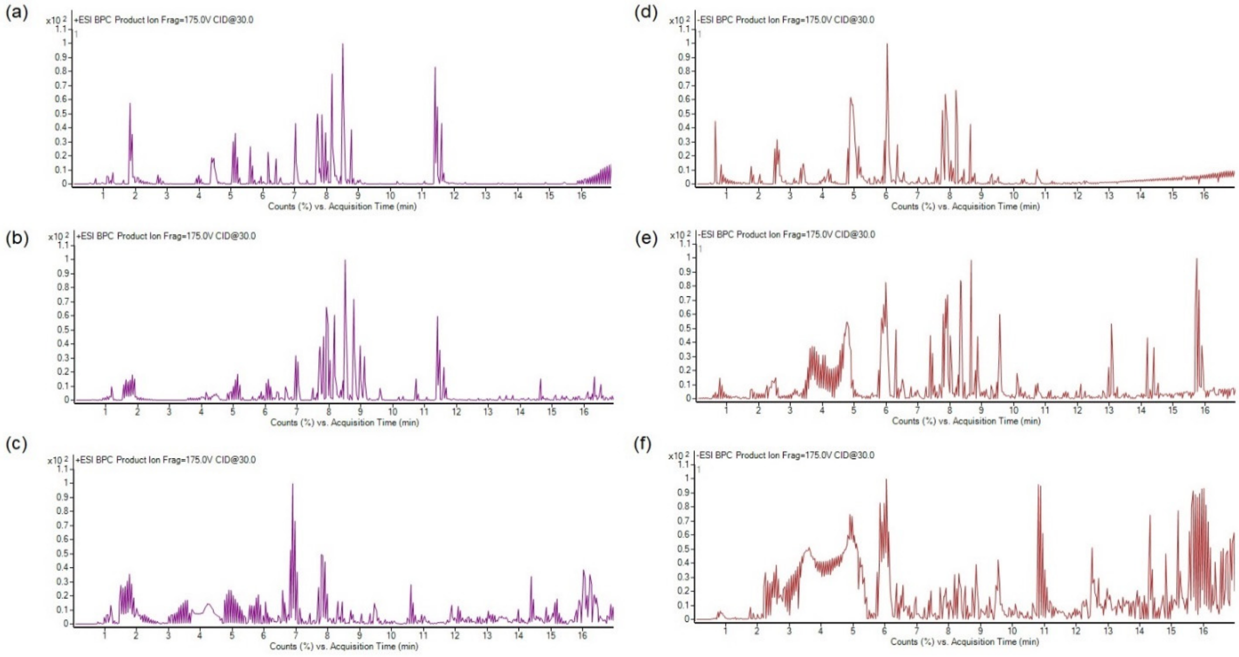

Figure S2. Base peak chromatograms of precursor ions and their MS/MS fragments (product ions) of: **(a)** Aqueous tea infusion (ATI); **(b)** Methanol/water (80/20 *v*/*v*) extract (MW); and **(c)** Methanol extract (M), in **positive** ionization modes; **(d)** Aqueous tea infusion (ATI); **(e)** Methanol/water (80/20 *v*/*v*) extract (MW); and **(f)** methanol extract (M), in **negative** ionization modes.

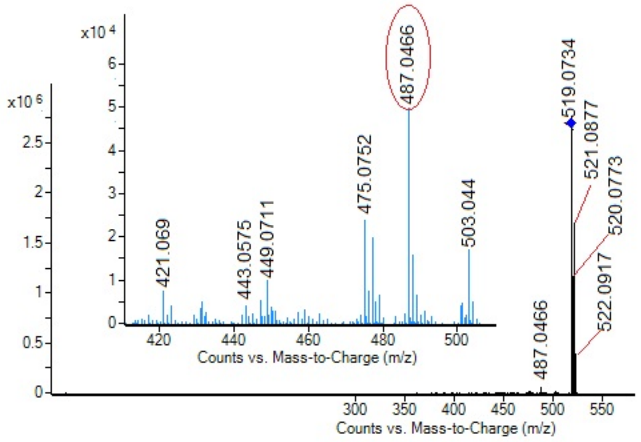

Figure S3. Characteristic MS/MS fragmentation pattern (collision-induced dissociation (CID) mass spectra), with major fragments of pseudohypericin (Agilent, Q-ToF, ESI(–), CE = 30 eV). The circled mass indicates the major fragment obtained by MS/MS fragmentation of this compound.

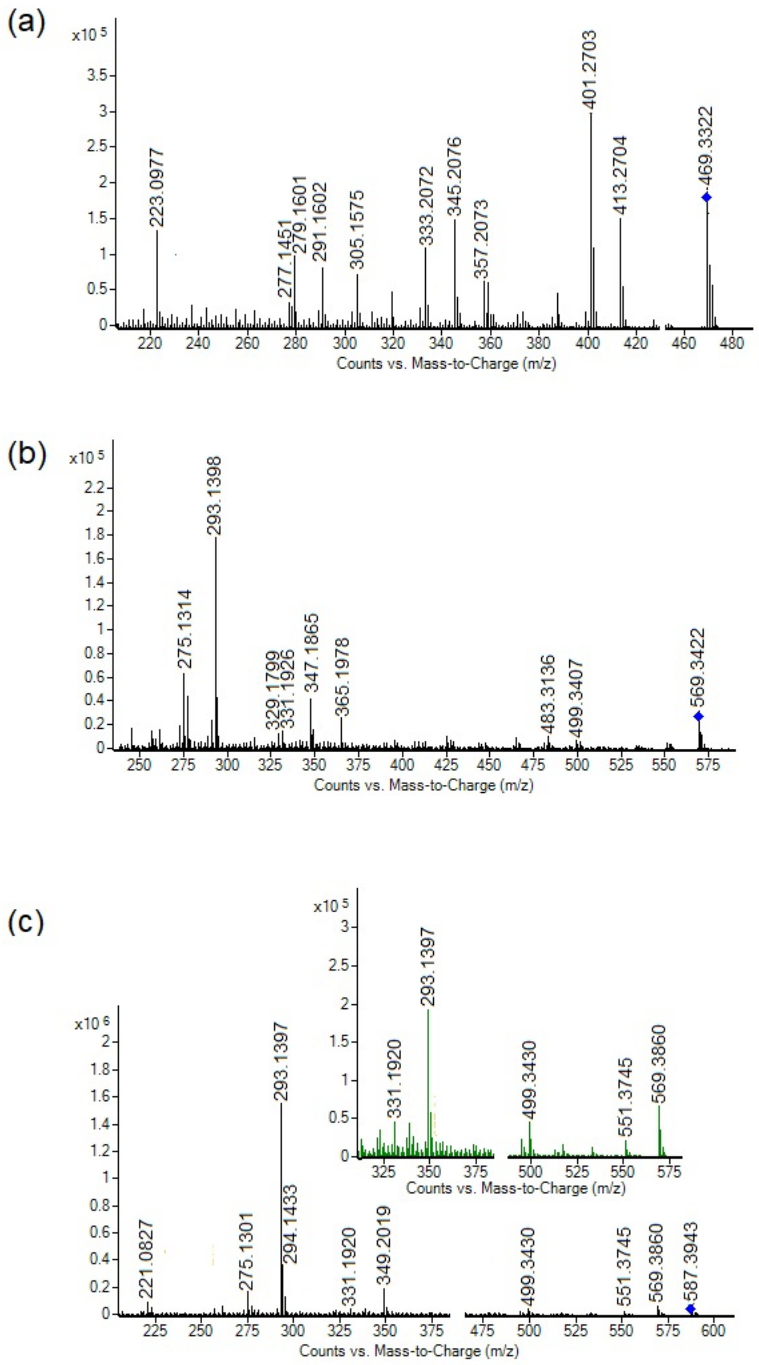

Figure S4. Characteristic MS/MS fragmentation patterns (collision-induced dissociation (CID) mass spectra), with major fragments of: **(a)** hyperfirin; (b) FPPAP derivative 3 (compound like Hyperformitin C or Hyperformitin D); **(c)** FPPAP derivative 6 (compound like Hyperidione F), (Agilent, Q-ToF, ESI(+), CE = 30 eV). Proposed fragmentation pathways of these tentatively identified compounds are presented in **Figure 5** (Manuscript).

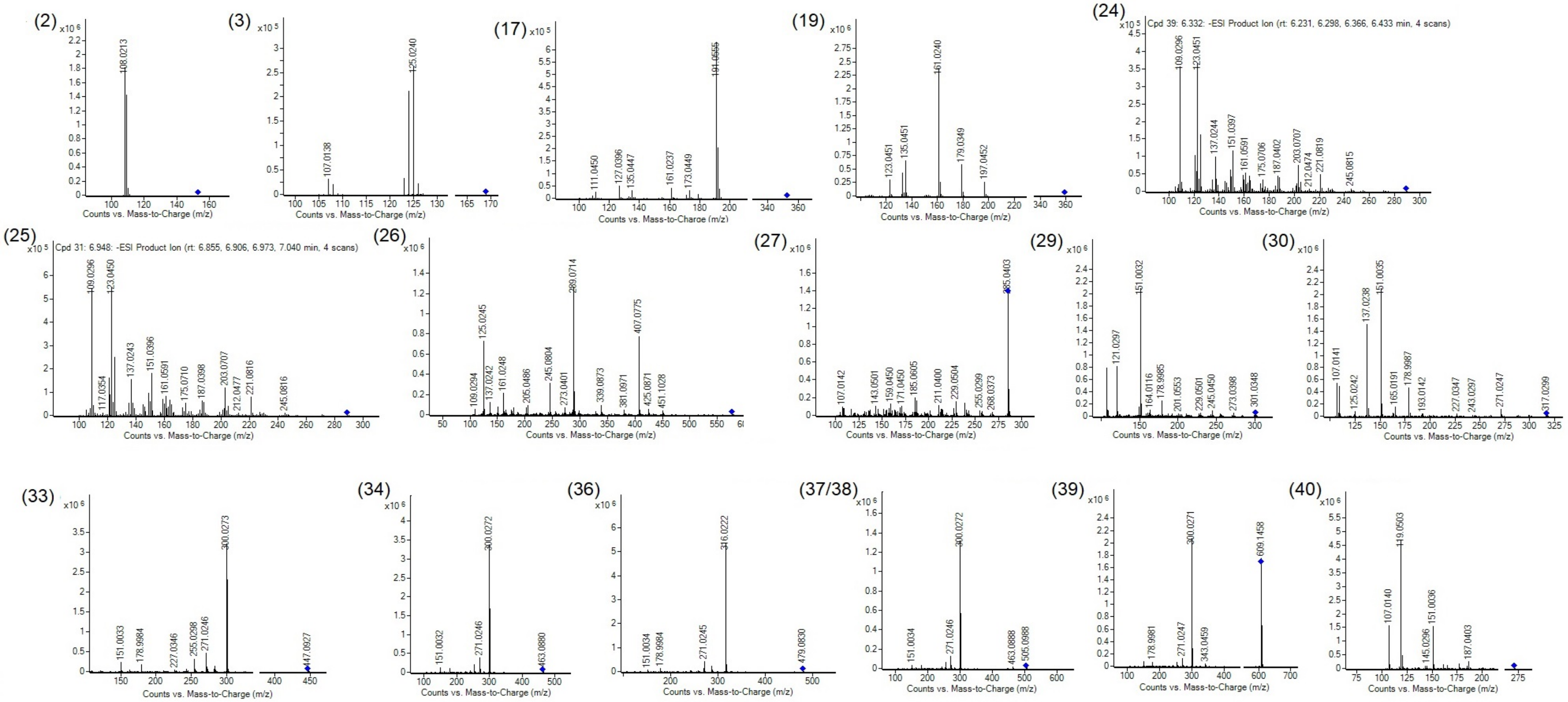

Figure S5. Fragmentation patterns (MS/MS spectra) of phenolic standards, which used for confirmation of phenolic compounds found in *H. perforatum* extracts. Compounds: (2) 3,4-dihydroxybenzoic acid (Protocatehuic acid) (Fluka, >99% purity); (3) Gallic acid (Chem Faces, >98% purity); (17) Chlorogenic acid (Chem Faces, >98% purity); (19) Rosmarinic acid (Chem Faces, >98% purity); (24) Catechin (Chem Faces, >98% purity); (25) Epicatechin (Chem Faces, >98% purity); (26) Procyanidin B2 (Chem Faces, >98% purity); (27) Kaempferol (Chem Faces, >98% purity); (29) Quercetin (Chem Faces, >98% purity); (30) Myricetin (Chem Faces, >98% purity); (33) Quercetin 3-*O*-rhamnoside (Quercitrin) (Chem Faces, >98% purity); (34) Quercetin 3-*O*-glucoside (Hyperoside) (Extrasynthese, >99% purity); (36) Myricetin 3-*O*-glucoside (Extrasynthese, >99% purity); (37/38) Quercetin 3-*O*-(6''-*O*-acetyl)-beta-D-glucopyranoside (Extrasynthese, >95% purity); (39) Rutin (Chem Faces, >98% purity); (40) Naringenin (Chem Faces, >98% purity).
